# Immunomonitoring of Monocyte and Neutrophil Function in Critically Ill Patients: From Sepsis and/or Trauma to COVID-19

**DOI:** 10.3390/jcm10245815

**Published:** 2021-12-12

**Authors:** Ivo Udovicic, Ivan Stanojevic, Dragan Djordjevic, Snjezana Zeba, Goran Rondovic, Tanja Abazovic, Srdjan Lazic, Danilo Vojvodic, Kendrick To, Dzihan Abazovic, Wasim Khan, Maja Surbatovic

**Affiliations:** 1 Clinic of Anesthesiology and Intensive Therapy, Military Medical Academy, Crnotravska 17, 11000 Belgrade, Serbia; ivoudo@gmail.com (I.U.); dragan2403@gmail.com (D.D.); snjezanazeba@hotmail.com (S.Z.); grondovic@gmail.com (G.R.); abazovic.tanja1@gmail.com (T.A.); 2 Faculty of Medicine of the Military Medical Academy, University of Defence, Crnotravska 17, 11000 Belgrade, Serbia; ivanivanstanojevic@gmail.com (I.S.); drlazics@gmail.com (S.L.); vojvodic.danilo@gmail.com (D.V.); 3 Institute for Medical Research, Military Medical Academy, Crnotravska 17, 11000 Belgrade, Serbia; 4 Institute of Epidemiology, Military Medical Academy, Crnotravska 17, 11000 Belgrade, Serbia; 5 Division of Trauma & Orthopaedic Surgery, University of Cambridge, Addenbrooke’s Hospital, Cambridge CB2 2QQ, UK; kendrick.to@doctors.org.uk (K.T.); wasimkhan@doctors.org.uk (W.K.); 6 Emergency Medical Centar of Montenegro, Vaka Djurovica bb, 81000 Podgorica, Montenegro; adzihan@gmail.com

**Keywords:** sepsis, trauma, COVID-19, monitoring, immunologic, biomarkers, immunosuppression, immunotherapy, therapy, critical illness

## Abstract

Immune cells and mediators play a crucial role in the critical care setting but are understudied. This review explores the concept of sepsis and/or injury-induced immunosuppression and immuno-inflammatory response in COVID-19 and reiterates the need for more accurate functional immunomonitoring of monocyte and neutrophil function in these critically ill patients. in addition, the feasibility of circulating and cell-surface immune biomarkers as predictors of infection and/or outcome in critically ill patients is explored. It is clear that, for critically ill, one size does not fit all and that immune phenotyping of critically ill patients may allow the development of a more personalized approach with tailored immunotherapy for the specific patient. In addition, at this point in time, caution is advised regarding the quality of evidence of some COVID-19 studies in the literature.

## 1. Introduction

Severe sepsis and/or trauma can lead to multiple organ dysfunction syndrome (MODS), which is a leading cause of death in intensive care units with mortality rates in excess of 50%. In addition to infection, the degree of immuno-inflammatory response also influences the outcome. While this response is essential for host defense against infection, left unchecked, it can lead to MODS. One way to view the immune response in this context is to envisage it as a negative feedback system with a detection and effector limb; in this regard, MODS can represent a perturbed negative feedback loop that results in uncontrolled and detrimental inflammation. Innate immune response is delivered through resident macrophages and polymorphonuclear cells (PMCs), via primary phagocytosis in addition to recruitment of granulocytes and monocytes. Monocytes, through rapid differentiation, are capable of expanding the macrophage population [1]; they can therefore be viewed as one of the detector and effector limbs. The immuno-inflammatory response to pathophysiological insult involves several other detections such as recognition of non-self as part of danger sensing mechanisms and effector functions, which include activation of the adaptive immune system [2]. Leukocytes, via pattern recognition receptors (PRRs), sense pathogen-associated molecular patterns (PAMPs) and danger-associated molecular patterns (DAMPs). The former of which include Gram-negative lipopolysaccharide (LPS), Gram-positive peptidoglycan, flagellin and RNA; the latter includes alarmins generated by tissue damage, heat shock proteins (HSPs) and high-mobility group box-1 (HMGB-1). These signals are transduced and amplified, resulting in the release of inflammatory mediators such as cytokines. It has been proposed that a persistent dysregulated state of inflammation, as seen in patients with prolonged ICU stays, should be viewed as a separate phenotype to traditional systemic inflammatory response syndrome (SIRS), as described above, which is followed by the compensatory anti-inflammatory response syndrome (CARS). This has been termed persistent inflammation, immunosuppression and catabolism syndrome (PICS) in order to encapsulate the trajectory of initial systemic inflammation followed by refractory immunosuppression and persistent inability to return to systemic rebalance [3].

Injury-induced immunosuppression encompasses trauma, ischemia–reperfusion injury and hemorrhage as an acute stress, during which cells die and intracellular molecules such as DAMPs are released into the extracellular microenvironment [4]. This can induce a strong immuno-inflammatory response without the presence of microorganisms. These molecules are not immunogenic per se, but upon release and transformation (oxidation, proteolysis), they acquire immunostimulatory properties and can induce deleterious inflammatory response. Depending on the microenvironment, DAMPs display different properties; adenosine triphosphate (ATP) is a pro-inflammatory DAMP, but if it undergoes hydrolysis, it produces adenosine, which is a potent inducer of immunosuppressive IL-10. DAMPs are also important in the process of tissue repair. Immunogenic cell death (ICD) is a process that involves changes to the composition of the cell surface and the release of the soluble mediator DAMPs that recruit and activate antigen-presenting cells, e.g., dendritic cells. This is a specific type of apoptosis in immunocompetent cells that elicits an immune response against dead cells [5].

To adequately assess the extent of an immuno-inflammatory response to pathophysiological insult, the use of conventional inflammation biomarkers can be seen as limited due to several reasons. First, the detection of peak levels of cytokines is difficult due to their short half-life and their role in a complex immuno-inflammatory cascade; this introduces a temporal issue. Studies of neutrophil and monocyte function in critically ill patients with sepsis and/or trauma may however act as a more accurate indicator.

As a sequela of sepsis, patients often suffer from chronic immunosuppression, which is attributed to an anti-inflammatory response that is triggered by low-grade inflammation. Long-term outcome studies have shown that sepsis survivors suffer from impaired immuno-inflammatory response to recurrent infections and increased mortality [6,7,8]. A macroscopic postmortem study of 235 surgical intensive care patients with sepsis found that 76.6% had an unresolved focus of infection [9].

As there is often no clinical sign of immunosuppression in critically ill patients, biomarkers of cell function determined by flow cytometry can identify patients who are, for the most part, deeply immunosuppressed and can benefit from immunostimulation [10,11]. There is an interest in novel therapeutic approaches to stimulate the immune function in patients with sepsis; these include interleukin-7 (IL-7), granulocyte–macrophage colony-stimulating factor (GM-CSF) as well as antibodies against programmed cell death protein 1 (PD-1) and programmed death-ligand 1 (PD-L1). Clinical trials for these therapeutic options should aim to evaluate immune status and to stratify patients through the use of immune function biomarkers in order to ascertain the degree and likelihood of benefits from therapy. In such cases, a biomarker with an appropriate half-life as well as a test that allows adequate sampling frequency is essential, as septic patients can be in a state of severe inflammation and/or immunosuppression at various different time points throughout illness.

While there is a significant genetic influence over the underlying cause, micro-organisms, and the eventual outcome of sepsis [12], there is evidence of a large non-heritable component that contributes to determining outcome. One study attempted to observe heritable versus non-heritable factors by performing a systematic analysis of 210 healthy twins from 8–82 years of age. The study measured more than 200 serological parameters that included cell population frequencies and cytokine responses. The study found that 77% of the parameters were greatly influenced (at >50% of variance) and 58% were almost completely determined (at >80% of variance) by non-heritable influences. A number of these parameters displayed more variability with age, illustrating the temporal cumulative effects of environmental exposure over the course of a lifetime. A divergent response to influenza vaccination in twins was also observed in the study, thus alluding to the immune system in healthy individuals as being largely reactive and adaptive [13]. As the population of septic, critically ill patients are increasing in age and number of co-morbidities, it stands to reason that they will develop a unique inflammatory profile given the cumulative environmental exposure over time; as such, it would be sensible to consider their serological response on a case-by-case basis.

Additionally, sustained immunosuppression is not specific to sepsis, and may be induced every time the body develops a significant inflammatory response to an insult. Monneret and Venet proposed the use of the more generic term of injury-induced immunosuppression (IAI) [10]. In the clinical setting of sepsis, as in many others, it is clear that one size does not fit all and that immune phenotyping of patients may eventually allow the more personalized approach, namely “precision medicine” [14].

Perturbed immune regulation in sufferers of COVID-19 emphasizes the importance of an appropriate immune response in critically ill patients [15]. Better understanding of severe COVID-19 immunopathogenesis will therefore improve survival of this patient population.

## 2. Elements of Immunopathogenesis and Molecular Signature of Sepsis- and Trauma-Induced Multiple Organ Dysfunction Syndrome—MODS

### 2.1. Sepsis-Induced Multiple Organ Dysfunction Syndrome

Sepsis is one of the first illnesses to be described. The term “sepsis” derives from the ancient Greek term “σῆψις, i.e., sipo” (“make rotten”). This term was used by Hippocrates around 400 BCE to describe the process through which infected wounds become purulent [16]. It took over two millennia to shift our focus onto the role of the host response, and not only on the pathogen itself, in understanding the pathogenesis of sepsis. It is estimated that there are 30 million cases of sepsis and six million sepsis-related deaths worldwide each year [17,18,19,20,21,22,23]; thus, it is an area of intense medical research.

The dichotomous role of neutrophils in inflammation and infection is well known. These cells play a crucial role in defense against infection; conversely, excessive activation of neutrophils can elicit tissue damage. This is one of the mechanisms that underlies sepsis-induced MODS [24]. Biomarkers of neutrophil activation may predict MODS in critically ill patients with sepsis [25]. Plasma concentrations of heparin-binding protein (HBP), myeloperoxidase (MPO), IL-6 and IL-8 appear to be correlated with emergence of the first sepsis-associated organ dysfunction. MPO and HBP become elevated at a median of 12 h before the first organ dysfunction and can remain elevated for up to 24 h, unlike two proinflammatory cytokines with rapid increase and decrease. Therefore, MPO and HBP, as markers of early prolonged neutrophil activation, are not as prone as IL-6 and IL-8 to the effect of sample timing and may be clinically more applicable. Circulating monocytes are also an important limb of innate immunity and are among the first cell-types to respond to pathogens. In a recent study of monocyte subsets in 42 critically ill patients with septic shock, [26] it was demonstrated that, in early deceased patients, the frequency of classical monocytes (within 12 h of admission) was significantly decreased, while frequency of intermediate monocytes was statistically highly significantly increased, in comparison with patients who survived past the fifth day of ICU stay. Therefore, myeloid cells are a current focus in research and in the clinical setting. Within the framework of complex immune cell function regulation, polarization of macrophage function is an important element. Classically activated macrophages (M1), initially in inflammatory response, can undergo reprogramming to an alternatively activated (M2) phenotype. This contributes to secondary immunosuppression during sepsis. Mitochondria in immune cells are also crucial for immunity. Mitophagy refers to selective degradation of mitochondria by autophagy. Mitophagy in monocytes/macrophages of critically ill patients was investigated by Patoli et al. who demonstrated that, in this patient population, mitophagy was inhibited in blood monocytes of septic in comparison with non-septic patients. They concluded that the inhibition of mitophagy is a physiological process contributing to myeloid cell activation [27].

MODS is the clinical consequence of a dysregulated, disproportionate immuno-inflammatory response to various stimuli, with a self-perpetuating cycle of neutrophil and macrophage activation [28]. Regardless of the trigger (sepsis and/or trauma), changes are profound at the genetic, molecular, subcellular and mediator levels. Transcriptome studies have revealed 363 differentially expressed genes during the acute inflammatory response in 36 major trauma patients who did develop MODS and those who did not. The number of differentially expressed genes decreased to 33 by the 24 h timepoint [29]. The role of T cells in the immune response during MODS development is shown in critically ill patients with sepsis [30] where Th17 (T helper cell) /Treg (Regulatory T cell) imbalance is related to MODS. Molecular signatures of MODS in critically ill patients reflect cellular dysfunction, hallmarked by mitochondrial dysfunction and endoplasmic reticulum stress [31]. In defense against microorganisms multiple, complex, and redundant processes are involved. For example, pyroptosis, which promotes release of cytokines [32]. Leukocytes release inflammatory mediators when activated via PAMPs and DAMPs; in response, there is overproduction of reactive nitrogen species (RNS) and nitric oxide. This is detrimental to electron transfer chain (ETC) function because this crucial pathway becomes irreversibly inhibited, leading to oxidative stress which is compounded mitochondrial DNA damage [33]. As a result, the energy metabolism of immune cells become defective; and various processes, including oxidative phosphorylation, are inhibited. This cascade of events manifests as immunosuppression [34]. In sepsis, unfolded or misfolded proteins accumulate in the endoplasmic reticulum (ER), this leads to oxidative and calcium dysregulation, resulting in ER stress [35].

The SEPSIS 3 definition for sepsis emphasizes the dysregulated host response to infection that leads to life-threatening organ dysfunction. The presence, severity and course of MODS determine the severity of clinical illness in the septic patients [36]. Cytokine imbalance is not only part of immunopathogenesis of sepsis and septic shock; it can also guide elements of therapy. A recent exploratory analysis of data from the Corticosteroid Therapy of Septic Shock (CORTICUS) trial showed that a low serum interferon-gamma (IFNγ)/IL-10 ratio was associated with increased survival in individuals treated with hydrocortisone whereas a high ratio was associated with better survival in the placebo group [37]. They concluded that IFNγ/IL-10 may become a suitable molecular maker to help inform the decision to use hydrocortisone in septic shock patients.

Another key component of immuno-inflammatory dissonance in critical care setting is activation of endothelial cells which may lead to endothelial dysfunction and glycocalyx destruction. This represents an incremental assault on virtually all organ systems. When this occurs, microvascular and tissue perfusion dysfunction, arteriovenous blood shunting, loss of vascular tone and procoagulant state are, nearly always inevitable [38]. Vasodilatation is the multifactorial consequence of acidosis on vascular smooth muscle, which leads to induction of nitric oxide production (NO). Loss of systemic vascular resistance may lead to complete vasoplegia; this might be initially compensated for some time by increased cardiac output but will be exacerbated by myocardial depression [39]. Fluid leak and tissue edema are ubiquitous: in the lungs, gas exchange is impaired, leading to acute respiratory distress syndrome (ARDS). Apart from microvascular derangements and tissue hypoperfusion, acute kidney injury may occur because of direct cytokine effects. Hepatic injury also occurs and this can contribute to coagulopathy. Specific forms of DAMPs, for example, bacterial and mitochondrial N-formyl peptides (NFPs) activate the innate immune system via formyl peptide receptors (FPR) which are present on immune and non-immune cells such as vascular endothelial cells [40]. The subsequent inflammatory response leads to endothelium barrier breakdown and the consequences described above. Novel approaches to try to protect the host from deleterious effects of imbalance between pro- and anti-inflammatory mediators include blood-filtering devices such as antibody-modified conduits (AMCs) that can remove specific cytokines in vitro. AMCs that use antibodies against human vascular endothelial growth factor A (VEGF-A) or TNF-α are able to remove specific cytokines from the blood in vitro [41].

The macrocirculation and microcirculation must be coherent to allow effective systemic hemodynamic-driven resuscitation that corrects organ perfusion and oxygenation. To further complicate matters, hemodynamic coherence is often lost during inflammation and infection [42]. Therefore, physiologic approaches are needed that allow the monitoring of the hemodynamic parameters and allow optimal oxygen delivery in shock resuscitation [43]. The importance of this principle is illustrated in the use of fluid challenge in critically ill patients [44]. Where there can be limited correlation between absolute changes in cardiac macrocirculation and small diameter vessels (microcirculation) in response to administration of a fluid challenge.

Host–pathogen interaction is complex, starting with recognition of pathogens by the host with consequent induction of inflammatory response via various Pattern Recognition Receptors (PRR) such as Toll-like receptors (TLR) [45,46]. Bacteria and cytokines have a multifaceted and intertwined relationship. One of the most interesting aspects of this relationship has been investigated by Meduri et al. for over two decades [47,48,49]. They demonstrated that IL-1β, TNF-α and IL-6 enhance bacterial growth in patients with sepsis-associated ARDS. They also found that at the onset of ARDS and over time, the level of these cytokine, both in plasma and bronchoalveolar lavage fluid—BAL, were significantly higher in nonsurvivors. They investigated extracellular and intracellular growth of *Staphylococcus aureus*, *Pseudomonas aeruginosa* and *Acinetobacter species* obtained from patients with ARDS, in response to graded concentrations of IL-1β, TNF-α and IL-6 in vitro. When the various bacteria were exposed to low concentrations of each proinflammatory cytokine (10–250 pg values equivalent to those in ARDS survivors), bacterial growth was not promoted, and monocytes were efficient in killing ingested bacteria. Conversely, when the bacteria were exposed to higher concentrations of cytokines (values equivalent to those in ARDS nonsurvivors), bacterial growth showed a dose-dependent enhancement. It was then demonstrated that impaired intracellular bacterial killing in activated monocytes was associated with increased expression of cytokines, and enhanced monocyte killing function on exposure to methylprednisolone was associated with decreased IL-1β, TNF-α and IL-6 expression [50]. The downregulation of dysregulated systemic inflammation is important in accelerating disease resolution and in decreasing the risk of nosocomial infections [51]. A similar study investigated the association of IL-6 and IL-10 levels with mortality in patients with sepsis and septic shock [52]. They found that IL-6 and IL-10 levels were both independently associated with mortality, but that the balance of these inflammatory mediators (IL-6/IL-10 interaction) does not seem to impact either early, intermediate or late mortality in ICU patients with sepsis. However, the balance of proinflammatory and anti-inflammatory mediator response may not be reflected by analyzing two pleiotropic cytokines [53], as IL-6 can be both proinflammatory and anti-inflammatory, depending on the context.

MODS can be considered a heterogeneous syndrome. Another important player in both organ and immune dysfunction is activated complement protein C5a, which exerts deleterious effects on organ systems as well as suppressing antimicrobial functions of key immune cells [54]. In polymicrobial sepsis, plasma products of complement activation in plasma including C5a anaphylatoxin and its receptors C5aR1 and C5aR2, are closely followed by extracellular histones that carries strong proinflammatory and prothrombotic activity [55]. In animal model of sepsis, both complement activation products and extracellular histones cause cell injury and multiple organ dysfunction. Neutralization of C5a through antibody or knockout prevents extracellular histones and the subsequent organ failure in septic mice.

An intricate network of immune cells is activated by PAMPs and DAMPs. Impaired macrophage function is considered to be one of the most important causes of immune paralysis and can contribute to organ dysfunction and lethal outcome in sepsis. One in vitro study demonstrated that endogenous purine ATP facilitates the killing of bacteria that cause sepsis by macrophages via P2X4 receptors (P2X4Rs) [56]. Given that extracellular levels of ATP are increased in sepsis, P2X4Rs might be promising therapeutic targets.

B cell responses are altered and are oriented toward an exhausted-like/immunoregulatory profile during sepsis-induced immunosuppression [57]. Natural killer (NK) cells are large granular lymphocytes, acting as coordinators of early responses to bacteria through production of interferon (IFN)-γ which amplify the antimicrobial functions of myeloid cells. Conversely, if excessive NK cell activation occurs, production of IFN-γ will increase and this can result in organ injury and dysfunction [58]. Extracellular vesicles (EVs) which are 30 nm to several µm in size, are released from immune cells on activation and apoptosis. EVs express membrane epitopes that are specific to their parental cells. There is speculation that EVs act as mediators in sepsis, both as friends and foes. Their role in systemic inflammation mostly depends on origin and the cargo they carry, which makes them potential candidates for drug delivery [59].

Inflammasomes are large, intracellular multiprotein complexes and may play a role in sepsis. They detect and respond to a number of PAMPs, including bacterial flagellin, and DAMPs, such as uric acid crystals. Apoptosis-associated speck-like protein containing a caspase-recruitment domain (ACS) is a key component of the inflammasome. When inflammasomes are activated and assembled, ASC moves from its diffuse distribution in the cytoplasm into a single speck that serves as a supramolecular signaling platform. These interesting structures promote the maturation of the pro-inflammatory cytokines IL-1β and IL-18. When evaluating ASC–speck formation in monocytes during the first week of sepsis in patients, the highest number of ASC–speck**^+^** monocytes can be detected on day 6–7. Survival analysis shows that patients with lower numbers of ASC–speck**^+^** monocytes (<1650 cells/mL) on day 6 has greater risk of mortality [60].

One of the mediators of chronic immunosuppression in sepsis is vagal activation [61]. Enhanced vagus nerve tonic activity results in an immunosuppressed phenotype in patients who survive sepsis. Since cholinergic tone can be pharmacologically modulated, targeting this process may be a novel therapeutic approach to prevent latter infections in these immunocompromised patients.

Long-term sequelae of sepsis immunology are becoming the focus of attention. In a study by Rodriguez-Rosales et al., long-term immune effects of human experimental endotoxemia were investigated when healthy subjects were challenged with endotoxin (1 ng/kg) [62]. Twenty days post-endotoxin, flow cytometry revealed, among other things, increase in absolute numbers of intermediate monocytes with lower human leukocyte antigen–DR isotype—HLA-DR expression. Long-term host immune response trajectories (up to 12 months) were investigated in a cohort of 483 hospitalized sepsis survivors [63]. Approximately 25% of these individuals had elevated C-reactive protein (CRP) at 12 months and about 50% of them had elevated soluble PD-L1 (a marker of immunosuppression). This suggest that persistent elevation of inflammation and immunosuppression markers is common up in sepsis survivors and may be associated with poor long-term outcomes. A recent review discussed syndrome of chronic critical illness (CCI) which encapsulate sepsis patients who survive the early “cytokine or genomic storm”, but then fail to recover fully, and progress to a persistent manageable organ dysfunction state that requires prolonged intensive care [64]. It is suspected that as many as one third of sepsis survivors develop CCI which is in part due to a maladaptive host response to processes mediated by PRRs. CCI is characterized by exhaustion and atrophy of T cells, expansion of suppressor cell function as well as chronic inflammation and dysregulated myelopoiesis. Authors proposed that PICS in survivors of critical illness represent a unique immune endotype, with persistent release of DAMPs and PAMPs from secondary infections.

Aging of the population predisposes to the development of both CCI and PICS. The elderly patients are more susceptible to sepsis and are at greater risk of mortality. This is, in part, result of immunosenescence and a marked decline in cell-mediated and humoral immunity that is seen with increasing age [65,66].

For the past two decades, attention to sepsis has been intensified because of growing recognition that it is one of the most common and lethal conditions we face (approximately 50 million people worldwide annually), whether as a patient, provider, hospital or public health agency [67]. Therefore, early prediction of sepsis is of utmost importance in order to provide optimal care at an early stage. Recent implementation of soft-computing and machine learning techniques can illustrate how complex and difficult this task is [68]. Both researchers and clinicians are acutely aware of the complexity and heterogeneity of sepsis which is a significant impediment to adequate treatment. Thus, quite an effort has been undertaken to identify subgroups of sepsis patients who represent distinct functional endotypes based on measurable genetic and biologic differences [69]. Endotyping may also identify individuals unlikely to benefit, or more likely to be harmed, by specific therapies. Sepsis is heterogeneous syndrome, characterized by a vast set of clinical and biological features [70], combinations of these features may represent previously unrecognized groups, or sepsis subclasses with different risks of outcome and response to a given treatment. The authors of one study established a machine learning model to classify sepsis into different immune endotypes based on transcriptomics data [71]. They identified two immune subphenotypes associated with sepsis and termed them immunoparalysis and immunocompetent endotypes. They also found that percentages of M0 macrophages, M2 macrophages, naïve B cells and naïve CD4 T cells were associated with cumulative mortality at 28 days. More than a decade ago, we investigated polymorphisms of genes encoding tumor necrosis factor-alpha, interleukin-10, cluster of differentiation-14 (CD14) and interleukin-1ra in critically ill patients [12]. Some of the polymorphisms were significantly associated with outcome, for instance. Epigenome-wide methylation analysis of whole blood DNA samples from a cohort of 66 septic and 68 non-septic critically ill patients on day 1 of ICU admission [72] and weighted gene co-expression network analysis was performed. It showed DNA co-methylation modules associated with severity of illness, need for vasopressors, and length of stay.

Understanding of the heterogeneity in the individual host response to infection is necessary for effective targeted sepsis therapy. Other studies investigated this heterogeneity by defining the variation between individuals in the transcriptome of patients with sepsis [73]. Transcriptomic analysis of peripheral blood leukocytes suggests the presence of two distinct sepsis response signatures (SRS1 and SRS2). SRS1 (detected in 41% patients) identifies individuals with an immunosuppressed phenotype that include features of impaired antigen processing ability and endotoxin tolerance, T cell exhaustion, as well as downregulation of human leucocyte antigen (HLA) class II. SRS1 was associated with significantly higher mortality in comparison with SRS2.

NF-κB (nuclear factor kappa-light-chain-enhancer of activated B cells) is a protein complex with a number of functions including control of DNA transcription, production of cytokines and cell survival [74]. This mediator was initially discovered in 1968 by Sen and Baltimore who identified its role in the transcription of immunoglobulin κ-light chains in B lymphocytes. One study demonstrated that sepsis patients display a reduced ability to activate NF-κB in multiple cell types [75]. Intensive care unit (ICU)-acquired infections (IAI) result in a longer hospital and ICU stay, as well as increased costs and mortality. To identify patients at risk of IAI, authors of one study evaluated the association of the systemic mRNA expression of two biomarkers of host response, CD74 (cell surface receptor for the cytokine macrophage migration inhibitory factor—MIF) and IL-10, with IAI in a large number of ICU patients [76]. They found that immune monitoring using these two immune biomarkers could be appropriate for the identification of IAI risk in ICU patients. This suggested that immune profiling of critically ill patients can be integrated through a multimodal real-time diagnostic work-up of IAI [77]. Investigating features of the immune response in sepsis, as potential biomarkers, is challenging because of the temporal effects: over time there are differences between patients (interindividual) as well as within the same patient (intraindividual) [78]. Functional immunity changes cannot be adequately assessed by routine non-specific inflammatory biomarkers (CRP, procalcitonin). Thus, sepsis biomarkers are still much in focus of numerous investigations [79,80,81,82,83,84,85].

### 2.2. Trauma-Induced Multiple Organ Dysfunction Syndrome

Trauma is the third leading cause of mortality worldwide as well as the first cause of fatality and disability in those younger than 45 years of age [86]. Later deaths are result of development of MODS and infections in trauma patients who are profoundly immunosuppressed; that occurs in 45% of severe trauma patients. Trauma-induced MODS and immunosuppression are, for the most part, result of innate immunity activation. DAMPs, normally hidden from the immune system, are abundantly released after severe musculoskeletal injury; through binding to cell surface and intracellular neutrophil receptors (PRRs for instance), they generate systemic inflammation. Mitochondria, organelles of bacterial origin, are important regulators of inflammatory response and are a platform for PRR signal transduction [87]. Spatial proximity of mitochondria and inflammasomes in perinuclear regions enables modulation of inflammasome by these organelles. Mitochondria share some molecular traits with bacteria; normally these components are not ligands for PRRs, but during cellular damage they are released and do act as DAMPs. These include mitochondrial DNA, large quantities of extracellular ATP, cardiolipin (normally found only in the inner mitochondrial membrane) and formyl-peptides. When innate immunity is unsuccessful in clearing DAMPs and/or PAMPs, adaptive immunity is activated. Mitochondrial metabolic states within synapse between dendritic cells and lymphocytes are able to polarize adaptive immunity: glycolytic metabolism is associated with proinflammatory, whereas oxidative metabolism is associated with anti-inflammatory response. The role of mitochondrial metabolism in dendritic cells therefore induces either pro- or anti-inflammatory differentiation of T helper cells. Given the fact that underlying mechanisms of trauma-induced MODS are not yet fully elucidated. Aswani et al., investigated whether mitochondrial (mt) DNA, released after various degrees of tissue damage and hemorrhagic shock, is sufficient to induce MODS in a rodent model [88]. mtDNA, similar to bacterial DNA, has large quantity of highly stimulatory unmethylated CpG DNA motifs, which are ligands for TLR-9 and will trigger inflammation. The authors demonstrated that release of mtDNA is sufficient for MODS development and they showed that neutralizing this mediator, as well as nuclear DNA, with the nucleic acid scavenging polymer, hexadimethrine bromide (HDMBr) is able to rescue from MODS. They concluded that it could have utility in treatment of human trauma-induced MODS.

Alarmins, which are DAMPs released after trauma include all nucleic acids, HMGB1, HSPs and S100 proteins. These mediators activate multiple receptors and signaling systems such as PRRs, Receptor for Advanced Glycation Endproducts (RAGE) and Triggering Receptor Expressed on Myeloid cells-1 (TREM-1). DAMPs activate neutrophils and dendritic cells; thus, both the innate and adaptive immunity are set in motion. Posttraumatic immunosuppression renders trauma patients susceptible to secondary infection. The role of DAMPs in that process have been investigated in adult trauma patients. A study reported an inverse relationship between levels of HSP70 and nuclear DNA on one hand and HLA-DR expression conversely. DAMPs also induce long-term endotoxin tolerance. Via TLRs, DAMPs may also induce epigenetic alterations [89]. These gene-specific chromatin modifications are associated with transient silencing of various classes of genes, including pro-inflammatory mediators [90]. HMGB1-RAGE signaling results in functional exhaustion of mature monocytes and lymphopenia; this is the hallmark of immune suppression following extensive brain ischemic injury [91]. DAMPs can induce immunosuppression without a preceding inappropriate inflammatory response. The endogenous purine nucleotides are major regulators of the inflammatory response [92]. Adenosine is a catabolite of ATP, and during inflammation it signals by binding and activating purinergic receptor. High amount of adenosine released after trauma may directly induce Th2 response [93].

Patients suffering from multiple traumas often require massive blood transfusion; thus, it is important to bear in mind that red blood cells contain DAMPs and promotes the formation of the inflammasome [92]. Potent DAMPs that may be released by red blood cell lysis include haem, HSPs, such as Hsp70, IL-33 and Adenosine 5’ triphosphate. Hemolysis represents a major inflammatory trigger [94]. IL-33 is expressed in the nucleus of epithelial cells and is released into the extracellular space following tissue damage. It has been shown to initiate the Th2-polarizing function of dendritic cells and stimulates the secretion of anti-inflammatory cytokines [95]. After tissue injury, massive DAMPs release leads to overwhelming systemic inflammation and early MODS; in addition, these mediators may lead to immunosuppression; thus, the severely injured are prone to secondary infection and late MODS. Plasma mtDNA is associated with the evolution of systemic inflammation, MODS, and increased mortality in severely injured patients [96]. HMGB-1 can activate alveolar macrophages to produce proinflammatory cytokines and induce acute lung injury (ALI) through TLR-4. Alteration in tight junction and increased permeability leads to interstitial lung edema [97]. DAMPs and EVs can activate innate immune receptors and coagulation cascades, and this leads to an inflammatory response and blood coagulation. Several immunothrombotic agents play a role in promoting inflammation and activation of coagulation, these include extracellular DNA, HMGB1, the S100 family of intracellular low-molecular-weight calcium-binding proteins and histones [98]. Histones are cationic nuclear proteins that packages DNA into nucleosome. Extracellular, circulating histones, released as DAMPs after trauma, express direct cytotoxicity to both epithelial and endothelial cells by altering membrane permeability and causes calcium influx. This is associated with post-traumatic ALI [99]. Another DAMP, N-formyl peptide is released from the from mitochondrial matrix and is a well-known leukocyte chemoattractant which promotes chemotaxis of neutrophils to regions of sterile inflammation. EVs contain cellular cargo-like proteins, DNA and RNA and play an important role in intercellular communication. However, these interesting structures also carry various immunothrombotic mediators such as mtDNA, HMGB1 or HSP, depending on their origin. Exosomes are smaller than 0.1 µm in size and originate from multivesicular bodies (MVBs) [100]. EVs can transfer their cargo by endocytosis, phagocytosis, and micropinocytosis as well as membrane fusion [101]. Microparticles, released from endothelial and circulating cells following sepsis-induced microvascular injury, can contribute to endothelial dysfunction, immunosuppression and MODS [102]. Polytrauma or invasive surgery will produce DAMPs and EVs that cause microinjury and de novo release of immunothrombotic DAMPs and EVs in distant organs, thereby promoting post-traumatic MODS [98].

Trauma-induced DAMPs, as well as PAMPs, may trigger assembly of inflammasomes that are intracellular multiprotein complexes. These were initially described in 2002 as caspase-1 activating multiprotein complexes [103]. Initial tissue damage, blood loss and subsequent secondary injuries will lead to local and systemic release of DAMPs. Recognition of these mediators by the innate immunity triggers both excessive inflammation (which propagate remote, secondary tissue damage) and immunosuppression (which may contribute to secondary post-traumatic infection and sepsis); contributing to MODS and increased mortality. In trauma, mechanical tissue injury and blood loss are associated with secondary ischemia/reperfusion (I/R), hypothermia, hypoxia, coagulopathy and neuroendocrine disorders. The consequences of these range from cell stress to cell death [104,105]. Inflammasomes are named after their intracellular receptor, including nucleotide-binding oligomerization domain-like receptors, or NOD-like receptors (NLR), Absent in melanoma 2 (AIM2)-like receptors—ALR, retinoic acid-inducible gene-I-like receptors (RLR) or pyrin [106]. Specific roles of inflammasomes, for example as an intra-cytosolic sensor detecting mostly intracellular stimuli, provide another means of activation, through recognition of specific ligands to the sensing of intracellular disturbances. Regardless of the stimulus, activated inflammasome allows the caspase-1 dependent cleavage of pro-IL-1β and pro-IL-18 [107]. DAMPs are crucial part of the pathogenesis of trauma-induced MODS and support a vicious cycle of injury [108]. Both inflammasome-mediated pro-inflammatory release and pyroptotic cell death promote the initiation, enhancement and propagation of trauma-induced inflammation [109]. Pyroptosis features include cytoplasm swelling and cell membrane destruction as well as release of intracellular contents into extracellular space, thus contributing to sterile inflammation [110]. Diffuse activation of endothelium in an organ-specific manner is set in motion after systemic release of DAMPs [103]. Investigation of in vivo mouse model of hemorrhagic shock demonstrated NLRP3 activation in lung vascular endothelial cells, enhancing the proinflammatory response via pyroptosis and IL-1β release [111]. Although anucleate, platelets have functional translational material associated with mRNA transcripts, including IL-1β; thus, these cells can assemble functional NLRP3 [103]. Platelets express various immune receptors, cell surface adhesion molecules and many immunomodulatory mediators contained in preformed granules. these cells adhere to endothelial cells and leukocytes to form aggregates when activated by circulating DAMPs [103,112]. In the setting of tissue damage, platelets facilitate leukocyte activation and adhesion to post-ischemic microvessels. They also modulate degranulation and phagocytosis of neutrophils [103]. Ischemia-reperfusion will compound tissue injury; at cellular level, reperfusion triggers enormous production of ROS, calcium overload and mitochondrial dysfunction. This chain of events can end in cell death. During I/R, the NLRP3 inflammasome–IL-1β–IL-18 axis is crucial in organ-specific tissue injury, such as myocardial injury and renal necroinflammation, for example [113]. The NLRP3 and AIM2 inflammasomes are also important in hepatic I/R injury. Locally released DAMPs such as ROS, ATP or extracellular histones will activate inflammasomes in Kupffer cells [114]. Traumatic brain injury (TBI) will immediately damage tissue, while the pro-inflammatory innate immune response to neuro-injury, termed neuroinflammation, will additionally extend lesions via secondary cellular damage [115]. The NLRP1 inflammasome is assembled before neuron and other CNS cells stimulation; thus, it is of special interest in TBI as a crucial factor of induction and propagation of neuroinflammation [116]. TBI impacts peripheral cellular immune response via the hypothalamic–pituitary–adrenal axis, thus contributing to secondary damage to distant organs and susceptibility to infection [117]. The lungs are particularly susceptible to trauma-related ALI, being exposed to central venous blood conveying systemic DAMPs from injured tissues through pulmonary vasculature. ALI and/or ARDS leads to systemic and local activation of NLRP3 inflammasome [118]. Critically ill trauma patients often need mechanical ventilation (MV) and massive blood transfusion. MV-induced inflammatory lung injury may be consequence of cyclic alveolar stretch-induced activation of NLRP3 inflammasome; mechanism involves DAMPs: mitochondrial ROS generation and uric acid release [103]. The priming step of transfusion-related ALI—TRALI may be associated with NLRP3 inflammasome expression in various types of lung immune and endothelial cells, which constitute what is known as the “first hit”, while DAMPs from stored blood units with some degree of hemolysis, leading to the presence of heme or extracellular ATP may induce inflammasome activation and subsequent inflammation, resulting in a “second hit” [94]. Finally, dysfunctional inflammasomes in immune cells may be involved in post-trauma immunosuppression [119]. Authors of one study reported that NLRP1 gene expression following LPS stimulation is reduced in trauma patient monocytes. The decrease in mRNA levels of NLRP1 persisted over 10 days from admission to the emergency department [120]. In monocytes isolated from non-trauma patients, who had undergone cardiopulmonary resuscitation, levels of AIM2 gene expression as well as ability to release IL-1β were downregulated [121].

Trauma accounts for 10% of deaths and 16% of disabilities worldwide [122]. After major trauma, massive release of neutrophils occurs. Circulating neutrophils are dormant until activated by PAMPs and/or DAMPs., and when activated they carry out various functions including phagocytosis, degranulation, release of neutrophil extracellular traps (NETs), ROS and cytokines. These cells have altered functions and phenotypic markers because banded and even immature cells, such as metamyelocytes, enter circulation from bone marrow [123]. Authors of one study reported that major trauma is associated with subsequent delay of neutrophil apoptosis for at least 10 days, whether trauma victims developed sepsis or not [124]. Accumulation of activated neutrophils will lead to NETs formation in response to injury [125]. Surgery, as a form of controlled trauma, can, by itself, induce formation of NETs (elective total hip replacement for example). In these patients, it is a part of sterile inflammatory response mounted by innate immunity [126]. Trauma modulates neutrophil phenotypes and can lead to increased cell size and membrane plasticity, as well as modified shape (elongation). Neutrophil cell size can be significantly different between trauma survivors and nonsurvivors [127]. Distinct neutrophil subsets have been suggested to exist in trauma patients and in a human acute inflammation model, in which the hypersegmented CD62L^DIM^/CD16^POS^ subset can be separated from mature segmented neutrophils by multiplex proteomics comparison and immunosuppressive capacity [128,129]. Subset of neutrophils, predominantly CD11b^(+)/^Gr-1^(+)^/CXCR4^(hi)^ neutrophils recruited by vascular endothelial growth factor A-VEGF-A might be beneficial to repair the initial trauma impact. This subset of neutrophils delivers large amounts of the effector protein matrix metalloproteinase-9 (MMP-9), required for revascularization and functional reintegration [130]. Trauma has an impact on neutrophil migration; high levels of the neutrophil chemotactic factor IL-8 have been found in trauma patients [131]. Increased oxidative burst in neutrophils correlates with more extensive brain tissue injury by ROS [132]. Neutrophils are major producers of ROS which have been recognized as a component of NLRP3 activator in hepatic I/R injury [133]. NLRP3 is essential for acute sterile inflammation [134]. Newly formed ROS in injured tissue results in the migration and activation of more neutrophils [122], thus creating a vicious circle.

In the early phase after major trauma, surgical procedures should be carefully planned. Surgical procedures can be viewed as additional trauma load and constitutes a “second hit”. Authors of a recent pilot study investigated the immune status of trauma patients [135]. They used highly standardized systems to draw peripheral whole blood from seven polytraumatized patients with high injury severity score (ISS ≥ 32) and challenged it with bacterial LPS. In comparison with samples from healthy volunteers there was a significant decrease in the release of monocyte-derived mediators and surprisingly stable, unaltered or even increased concentrations of cytokines related to T cell maturation and function (IFN-γ, IL-2, IL-4 and IL-9). Levels of pro-inflammatory cytokines were reduced in response to LPS early after severe trauma. However, 24 h after injury, TNF response was not profoundly impaired. This led the authors to conclude that functional immune monitoring may be used to optimize the timing of necessary surgical interventions in severely injured patients. Another recent study focused on trauma-induced long-term alterations of immune response six months after major trauma event in 12 survivors [136]. CD4, CD8, CD14, PD-1, B and T lymphocyte attenuator (BTLA) cytotoxic T-lymphocyte-associated protein 4, TLR-2, -4, and -5, Dectin-1, PD-1L and HLA-DR expression were determined by flow cytometry. Cytokine release (IL-2, -4, -6, -10, and 17A, TNF-α, IFN-γ) was determined after stimulation of whole blood with LPS-, α-CD3/28, or zymosan. At the time-point of six months post-trauma, the overall immune responses were toward immunosuppression. They reported monocyte TLR-2 and TLR-4 suppression, for the first time six months after trauma which can also be observed during severe trauma [137]. It was also found that 6 months after trauma, there was no longer HLA-DR suppression, which is a feature characteristic of the early response to polytrauma. This may be explained by the short life span of classical and non-classical monocytes [138]. Following hospital discharge following traumatic injury, hospital-acquired infections remain a cause for post-discharge mortality [139,140]. An immunosuppressive phenotype, observed in neutrophils and monocytes, with impaired cytokine production after LPS challenge, is a common mechanism for trauma-induced MODS [141]. Therefore, it is important to explore ways of predicting the development of trauma-induced MODS, to allow detection as early as possible. Authors of one study measured a variety of inflammatory mediators from blunt trauma victims almost immediately after the event (within 24 h) to derive patient-specific “inflammation barcodes”. These barcodes can be used to predict development of MODS much more reliably than individual inflammatory mediators [142]. Trauma causes an abrupt transition from health to systemic physiological crisis. A recent study applied single-cell RNA sequencing to mononuclear cells from the peripheral blood and bone marrow in injured mice and trauma patients [143]. Transcriptomic analysis of leukocytes from severe trauma patients revealed a “genomic storm” with more than 80% of the leukocyte transcriptome altered during the first 28 days after major trauma. The greatest changes in gene expression in mice was seen in monocytes. After systemic injury, the monocytes gene expression pattern deviated from a steady state with similar changes in critical transcription factors. The changes in human CD14+ monocytes can be generalized into six signatures (SGs) with two trauma patient subtypes (SG1 vs. SG2) in the whole-blood leukocyte transcriptome in the first 12 h following injury. SG1 patients showed a longer recovery, more severe dysfunction in organs, and a higher number of complications compared with SG2. The two subtypes were also repeated for burn and sepsis patients suggesting a common immune response pathway.

Most clinicians consider medical and surgical patients to represent two varied groups, and that infection greatly affects the mortality in surgical patients [144]. Tissue damage and blood loss during surgical procedures will induce systemic inflammation. Contribution of anesthesia to immune modulation must also be considered. Opioids, such as the widely used remifentanil, are immunosuppressants and act via opioid receptors on leukocytes. One review [144] concluded that surgical infections are different from medical infections for a variety of reasons specific to surgical patients for example, due to a primed systemic inflammatory response caused by surgical insult, immediate postoperative immune suppression, anesthesia-induced immunomodulation, blood transfusion, I/R injury, etc. Thus, the course of surgical infections is more complex than medical ones. Authors of one study investigated immune response in a specific surgical setting, cytoreductive surgery (CRS) and hyperthermic intraperitoneal chemotherapy (HIPEC). They found that increased plasma levels of DAMPs (HSP70, HMGB1, S100A8/S100A9, S100A12, nuclear DNA, lactate dehydrogenase—LDH, which is a nonspecific marker of unscheduled cell death), were associated with immune suppression and postoperative infections [145]. CRS-HIPEC procedure caused excessive DAMP release. An increase in plasma HMGB1 levels was found to be associated with the decrease in HLA-DR expression in the aforementioned study. This is consistent with the findings of another study which included blunt chest trauma patients, where HMGB1 concentrations were associated with a higher risk for sepsis [146]. The amount of cell-free DNA, another important DAMP, is a prognostic tool for mortality as well as trauma severity and post-traumatic complications [147]. Burn victims are especially susceptible to infection. Neutrophil phagocytosis, oxidative burst capacity NET generation (NETosis), immature granulocyte (IG) count, plasma cell-free DNA (cfDNA) and plasma citrullinated histone H3 (Cit H3—a specific marker of NETosis) levels were measured up to one year following burn injury in 63 patients with burns to ≥ 15% total body surface area in an interesting study [148]. In addition, were measured. Neutrophil dysfunction, elevated IG counts as well as elevated plasma cfDNA and Cit-H3 levels were reported during septic episodes. All of the aforementioned measurements demonstrated potential as biomarker(s) of sepsis following burn injury. Neutrophil dysfunction may also actively contribute to the development of sepsis. Another interesting, recent study focused on surgical patients. Fresh blood samples revealed leukocytes with reduced viability in critically ill surgical patients. The authors investigated decreased leukocyte viability, the implications for leukocyte functioning and its clinical implications [149]. Non-viable neutrophils in vitro are referred to as fragile neutrophils in vivo. Overall neutrophil function was found not to be impaired in patients with fragile neutrophils, but these cells were associated with critical illness. Of the 11,871 patients, 75 (0.63%) had fragile neutrophils during hospitalization, and75.7% of these developed an infection, 70.3% required ICU admission and 31.3% died in hospital. Therefore, fragile neutrophils were mostly detected in surgical patients with recurrent or serious infections. Conversely, these cells were also observed in the absence of infection in patients who sustained high energy trauma and in patients with multiple or major surgeries (second hit). An advantage of this study is the usage of standardized, routine hematology analyzer [150], because manual work-up of blood samples leads to high number of apoptotic and necrotic neutrophils (up to 99%) due to in vitro manipulation which can easily affect results.

## 3. Programmed Cell Death 1 (PD-1)/Programmed Cell Death Ligand 1 (PD-L1) Expression on Monocytes

Circulating monocytes can be divided into three subsets according to the CD14 and CD16 antigen surface expression: CD14^++^ CD16^−^ (classical subset, constitute about 90% of the entire monocyte pool), CD14^++^CD16^+^ (intermediate subset) and CD14^+^CD16^++^ (non-classical subset). The last two subsets account for about 10% of circulating monocytes in healthy individuals [151].

PD-1 (CD279), first described by Ishida et al. in 1992, is a type I membrane protein of 268 amino acids. This cell surface receptor is a member of the extended CD28 family and is expressed, among other immune competent cells, on circulating monocytes (mPD-1). Its structure consists of an extracellular immunoglobulin superfamily IgV (Variable—V type) domain, a transmembrane region and an intracellular tail. The intracellular region of PD-1 receptor is made up of immuno-receptor tyrosine-based inhibitory motif (ITIM) and immuno-receptor tyrosine-based switch motif (ITSM). PD-1 protein is encoded by the *Pdcd1* gene that is located on chromosome 1 in mice and chromosome 2 in humans. Human and murine PD-1 proteins share almost 60% amino acid identity [152]. First identified in 1999, the activated PD-1 receptor generates a strong anti-inflammatory signal. Programmed death-ligand 1 (PD-L1) is a 40 kDa type 1 transmembrane protein also known as cluster of differentiation 274 (CD274) or B7 homolog 1 (B7-H1), and it has powerful immunosuppressive properties. PD-1/PD-L1, forms a co-inhibitory system and is considered an immune checkpoint molecule. This pathway appears to be especially important in sepsis-induced immunosuppression, as part of a negative feedback mechanism. PD-1 is expressed on activated T cells, natural killer (NK) cells and B cells. Its ligand, PD-L1, is expressed on both hematopoietic, nonhematopoietic cells and even tumor cells. It can also be found in parenchymal cells of organs including the heart, placenta, lung, liver, pancreas and kidney. PD-L1 has been implicated in organ injury during sepsis, especially intestinal and liver injury. PD-L1 plays a major role in the PD-1/PD-L1 pathway with inhibitory effects, while PD-1 is an auxiliary part of that process. The inhibitory immune checkpoint interaction often leads to T cell exhaustion. The ability of PD-1 to suppress T cell activation depends on the phosphorylation of the immunoreceptor tyrosine-based switch motif [11].

In septic patients, a pattern of increased PD-L1 expression on monocyte of has been confirmed in several studies [153,154,155], this is usually accompanied with decreased HLA-DR expression [153]. In a recent study, the relationship between PD-L1 expression on CD14^+^ monocyte (mPD-L1) and infectious complications in acute pancreatitis was evaluated. Sixty-three ICU patients with acute pancreatitis (AP) and 32 sex and age-matched healthy controls were enrolled in a prospective study. On days 1 and 3 following the onset of AP, PD-1 expression on peripheral CD4^+^ T cells, as well as PD-L1 and human leukocyte antigen-DR (HLA-DR) expression on CD14^+^ monocytes were measured. IL-10 levels were also determined. Percentages of PD-1 expressing CD4^+^ lymphocytes and PD-1L expressing CD14^+^ monocytes were found to be raised in patients with AP compared with healthy controls. Increased PD-1/PD-L1 expression was associated with a greater risk for infectious complications and increased plasma IL-10 levels. It was shown that an element of monocyte function, in particular the percentage of HLA-DR and PDL1 expression on CD14^+^ monocyte on day 1 was found to be an independent predictor of complication. The group concluded that PD-1/PD-L1 system plays an essential role in early immunosuppression, and that PD-L1 expression on monocytes may be a useful biomarker as indicated by the receiver operating characteristic curve (ROC) whereby PD-L1demonstrated a greater Area Under Curve (AUC) of 0.708 vs. HLA-DR AUC of 0.652, thus suggesting a commensurate and moderately superior diagnostic ability [156].

The association of monocyte PD-L1 expression after 3–4 days of sepsis with risk stratification and mortality was examined in another prospective cohort study [157] with29 healthy controls, 59 patients with sepsis and 76 patients with septic shock. Blood samples were obtained 3–4 days following systemic inflammatory response syndrome (SIRS). PD-1 expression was measured on circulating CD4^+^ T cells and CD8^+^ T cells while PD-L1 was measured on monocytes by flow cytometry. The study showed that only monocyte PD-L1 expression correlated to disease severity and consequently mortality. In particular, monocyte PD-L1 expression was an independent predictor of 28-day mortality in patients with septic shock. PD-L1 was the optimal marker for predicting mortality; a ROC curve analysis showed that patients with over 44.2% of monocytes expressing PD-L1 had a higher probability of death.

As part of the negative feedback system, immune checkpoint molecules act as negative regulators that modulate T cell responses. Co-ligation of T cell receptors (TCR) and PD-1 molecules induces an inhibitory signal in T cells that was characterized by cell cycle arrest, inability to proliferate and reduced cytokine synthesis, this effect is termed T cell exhaustion [158]. T cell exhaustion is mediated, in part, by PD-1/PD-L1 axis effects, this can be demonstrated in animal models by administration of antibodies targeting PD1 and PD-L1 which acts to prevent lymphocyte depletion, and consequently this significantly improved survival rates in septic mice.

The possibility of restoring immune response by using biologics to target this interaction has also been examined in septic patients [159]. The study evaluated the potential efficacy of blocking PD-1 and PD-L1 inhibitory pathways in sepsis, after extensive phenotypic and functional analysis of both innate and acquired immunity in critically ill septic and non-septic patients as well as healthy controls. Neutrophil and monocyte function were progressively diminished as sepsis persisted and this deterioration correlated with increased PD-L1 expression and with PD-1 expression on CD8^+^ T cells and NK cells. Importantly, blocking the checkpoint inhibitors PD-1/PD-L1 with antibodies restored function in neutrophil, monocyte, T cells, and NK cells, suggesting that this checkpoint could be acting as a key regulator of immune function under this particular setting in humans.

In patients who survive sepsis, there is speculation that there is some element of long- term immune impairment. This has been postulated to be the underlying reason for delayed death in patients who survive sepsis. The role that PD-1 plays in this phenomenon has been explored. One study observed that in the CD4^+^ T cells of eight sepsis survivors, PD-1 receptor density was found to be downregulated as compared with healthy controls. Conversely, B and T lymphocyte attenuator (BTLA) receptor expression trended toward upregulation. This suggests that in addition to PD-1, an alternate negative feedback pathway via BTLA could be responsible for immune dysfunction when considering sepsis survivors. The study found that long-term sepsis survivors had an increased number of clinically evident infections and low-grade inflammation based on standard inflammatory markers, additionally, cytokine production in response to stimulation appeared to be diminished in sepsis survivors. The sepsis survivors also demonstrated alterations in monocyte surface expression in pattern recognition receptors (PRR), most pronouncedly observed in decreased Toll-like receptor-5 (TLR-5). Investigation of PD-1L and HLA-DR expression on monocyte showed no significant differences between two groups in survivors of sepsis which is contrary to the case observed septic patients [160].

Recent studies have aimed to explore the variation in serum concentrations of soluble PD-1 and PD-1L in critically ill patients with sepsis and/or septic shock. One study assessed the kinetics of sPD-1 and sPD-1L in 30 septic ICU patients and 30 non-septic ICU [161]. sPD-1 and sPD-1L were found to be significantly higher in the septic group compared with the non-septic ICU group (17.7 vs. 4.5 pg/mL, *p* = 0.002; and 29.9 vs. 11.3 pg/mL, *p* = 0.02; respectively). Higher sPD-1L on day 3 following diagnosis of sepsis was associated with increased mortality. (16.7 vs. 3.0 pg/mL, *p* = 0.054) This was also observed in the total ICU cohort (14.9 vs. 2.7 pg/mL, *p* = 0.026). The correlation between the two immune checkpoint molecules was also significant at both days 1 and 3, suggesting that they can serve as a predictor early on. (*p* < 0.001, *p* < 0.001 and *p* = 0.004, respectively). Contrary to this, another study established that there were no differences in levels of sPD-1 or sPD-L1 between patients with sepsis when compared with healthy controls. No correlation was found between serum sPD-1 and sPD-L1 concentrations in patients with sepsis and lymphocyte surface expression [162]. In another prospective, single-center observational study undertaken in a surgical ICU, 86 consecutive patients admitted for septic shock of abdominal origin were observed. Fifteen plasma biomarkers (including sPD-1) were measured at ICU admission (86 patients), at ICU discharge (55 patients) and at one year after ICU discharge (46 patients). At ICU admission, concentrations of sPD-1 were found to be identical in controls and septic shock patients (0.05 ng/mL and 0.04 ng/mL respectively), 0% of patients had values measured outside the normal range. At the time of discharge from ICU, 95% of patients had abnormal sPD-1 values and one year later, 80 % of patients still had values (*p* < 0.0001) outside the normal range. However, there was no clear correlation between sPD-1 levels and ICU outcome. This study allowed the observation that increased immunosuppression at ICU discharge persisted for one year; while the level of sPD-1 was marginally lower, it remained abnormally elevated [163].

Another study found that sepsis survivors with hospital acquired infections who go on to develop chronic critical illness and persistent inflammation, immunosuppression and catabolism syndrome (PICS) are found to have greater levels of immunosuppressive proteins such as sPD-L1 [164]. The same group also sought to determine whether the incidence of secondary infections and immunosuppressive biomarker profiles of septic patients with chronic critical illness (CCI) differ from those with rapid recovery (RAP) after sepsis. The authors concluded that septic patients demonstrate clinical and biological features to suggest immunosuppression at the time of sepsis diagnosis. Those who developed CCI have a higher number of secondary infections and persistently deranged immune markers although measurements at the time of sepsis onset did not demonstrate a significant difference between subjects with RAP and CCI [165].

Soluble PD-L1 levels have also been investigated in the context of acute pancreatitis (AP), Chen et al. obtained blood samples from 56 patients with acute pancreatitis and compared this to a group of 21 healthy controls. Serum sPD-L1 levels as well as mHLA-DR were measured within 48 h following onset of acute pancreatitis. Authors demonstrated that sPD-L1 was significantly upregulated in patients with early AP, especially those with infectious complications, compared to healthy controls. Significant negative correlations were observed among mHLA-DR expression, lymphocyte count and sPD-L1 levels in AP. Multivariate regression analysis showed that sPD-L1 was an independent early predictor of infectious complications in AP [166]. PD-L1 expression appears to have some relationship with certain physical parameters, the influence of hypoxemia on immune response was investigated by Avendano-Ortiz and coworkers. They concluded that SaO_2_ levels on admission might serve as a potential marker for immune status, including PD-L1 expression [167].

Given the fact that immunosuppression has been a primary focus of sepsis research in recent years, it is obvious that negative costimulatory molecules such as PD-1 and PD-Ll are key elements of its pathophysiological mechanism [168]. The general immunosuppressive attributes of PD-1/PD-L1 axis implicate these immune-inhibitory check point molecules in various conditions, such as inflammatory diseases of blood vessels [169]. Other immune checkpoint ligands in sepsis have been investigated. For example, sialic acid-binding immunoglobulin-type lectins (SIGLECs) may play an important role in modulating the immune response in sepsis and serve as survival marker [170]. More research is needed to elucidate multifaceted immune dysfunction in sepsis [33].

## 4. Programmed Cell Death 1 (PD-1)/Programmed Cell Death Ligand 1 (PD-L1) Expression on Neutrophils

Although it has been shown that neutrophils, by expressing PD-L1, may inhibit proliferation of lymphocytes, to our knowledge, there is limited literature that explores this in the context of sepsis. In the literature available to us, we identified only three studies; one animal study, one that included both human and animal subjects, and one that included only human, all with low numbers of patients. One showed, in an animal inflammation model, that neutrophils in draining lymph nodes upregulate PD-L1 expression and can suppress T cell proliferation. The study emphasized the critical role of neutrophils in adaptive immunity homeostasis via a PD-L1 dependent mechanism [171]. One study was performed to determine the level of PD-L1 expression on neutrophils in 41 patients with severe sepsis as well as in six septic mice. They found that PD-L1 was significantly upregulated on neutrophils from both septic patients and mice. in addition, neutrophil PD-L1 was good predictor of outcome in patients with severe sepsis with AUC of 0.74 [172]. This is contrary to our results (unpublished data) from our investigation of 86 critically ill patients with secondary sepsis. We studied a broad panel of immune biomarkers on neutrophils and monocytes, among them was PD-L1 on neutrophils. There were no statistically significant differences in neutrophil PD-L1 expression in either of chosen time intervals (first and fifth day) between survivors and nonsurvivors. Yet, we found higher expression of this immune marker on the fifth day compared to day 1; this was statistically significant only in nonsurvivors. Finally, the third and most recent study demonstrated two new subsets of immature and dysfunctional neutrophils, distinguished by CD123 and PD-L1 expression, which defined as an early human blood signature of sepsis [173]. The authors enrolled 17 ICU septic patients, 12 non-infected post-cardiothoracic surgery patients, 11 healthy donors and five orthopedic surgery patients with bone marrow biopsies. The results indicated a statistically highly significant difference in neutrophil PD-L1 expression between the sepsis group and the other groups. In ICU patients with sepsis, PD-L1^+^ neutrophils were significantly more abundant. It is therefore obvious that further research in this area is warranted.

## 5. Human Leukocyte Antigen D-Related—HLA-DR Expression on Monocytes

Major histocompatibility complex (MHC) is a set of cell surface proteins crucial for recognition of foreign molecules by adaptive immune system. Human leukocyte antigen D-related (HLA-DR) is the MHC class II molecule expressed on most types of immune cells such as monocytes/macrophages, dendritic and B cells. HLA-DR expression correlates with immune cell activation and antigen presentation, a step that initiates the adaptive immune response. Conversely, a low level of HLA-DR expression is associated with an anti-inflammatory phenotype. In 1990, Hershman et al. first reported a decreased frequency in HLA-DR^+^ monocyte soon following trauma in healthy individuals. There is a plethora of influences that preside over the control of HLA-DR expression on immune cells. Their expression is up- and downregulated by pro-inflammatory cytokines such as interferon-gamma (IFNγ) and anti-inflammatory cytokines such as IL-10, respectively. Medication such as corticosteroids and catecholamines are also able to reduce HLA-DR expression. Monocytic HLA-DR (mHLA-DR) expression is a pivotal link between innate and adaptive immunity; thus, the key interplay of monocytes with T cells is often colloquially referred to as “immunological synapsis” [174]. The persistence and magnitude of mHLA-DR expression has been used as a global marker of immune function in critically ill patients since it was first proposed whereby, a low mHLA-DR serves as an indicator of monocyte anergy and is associated with lower tumor necrosis factor (TNF)-alpha and IL-1 production in response to bacterial insult [175].

Monneret et al. conducted one of the landmark studies which attempted to describe mHLA-DR expression as a predictor of mortality in septic shock patients [176]. The group explored whether a low mHLA-DR expression, as a biomarker of immunosuppression, is an independent predictor of mortality in 93 septic shock patients who survived the initial 48 h of septic shock. While mHLA-DR expression levels were not significantly different between survivors and nonsurvivors within the first 1–2 days, significant differences were observed at days 3–4 with increased percentage of HLA-DR positive monocyte in survivors (43%) as compared with nonsurvivors (18%). Multivariate logistic regression analysis showed that low mHLA-DR (<30%) at days 3–4 is an independent predictor of mortality in septic shock patients. The ROC curve demonstrated that 30% HLA-DR positive monocytes at days 3–4 is the best cut-off value for mortality prediction with an AUC of 0.76. Therefore, dynamic changes in mHLA-DR expression over time in the setting of sepsis are important in view of potential inter-individual variations.

Following that, the same group aimed to address whether low mHLA-DR expression was associated with an increased number of nosocomial infection (NI) after septic shock in 209 septic shock patients. mHLA-DR was measured at days 3–4 and 6–9 after the onset of shock, and patients were screened daily for the development of NI [177]. mHLA-DR at days 3–4 was found to be diminished in nonsurvivors (20%) versus in survivors (43%), a similar result to previous studies. In line with these findings, the mHLA-DR value expressed as Means of Fluorescence Intensities (MFI) was 33 in nonsurvivors versus 67 in survivors. At days 3–4, patients who went on to develop NI had lower MFI values (39 versus 65 in those without NI). ROC curve analysis revealed that an MFI value of 54 was the best cut-off value to predict NI development with a sensitivity of 68% and specificity of 62%. At days 6–9, best cut-off MFI value was 57 with AUC of 0.64 (sensitivity 66%, specificity 60%). The study demonstrated that mHLA-DR ≤ 54 at days 3–4, and ≤ 57 at days 6–9 remained independently associated with NI occurrence after adjustment for clinical confounders. The study concluded that persistent low mHLA-DR expression was an independent predictor of secondary NI development in septic shock patients.

There is an emerging body of evidence that immune biomarkers are essential to guiding immunotherapy and risk stratification on an individual basis. Functional assessment of the immune system using mHLA-DR expression may reflect the net sum of pro- and anti-inflammatory factors and, therefore, the actual inflammatory phenotype and the phase of sepsis as such, this can be a better choice than using single pleiotropic and redundant inflammatory mediators [178].

It has been suggested that utilization of a combination of several immune cell function markers provide benefit over interpretation of individual biomarkers alone in predicting risk for NI and outcome in critically ill patients. Conway Morris et al. demonstrated that a combination of three measures of immune cell function namely: neutrophil CD88, mHLA-DR expression and percentage of regulatory T cells were significantly predictive of susceptibility to developing NI [179]. In their previous study they showed that critically ill patients have significant dysfunction of neutrophils from peripheral blood, mediated predominantly by activated complement (C5a) [180]. A recent follow up study (INFECT study) has been completed by the same group, aimed at validating their results in a cohort of critically ill patients; in the setting of trauma, sepsis and post-surgical complications which all bear similarities in the innate and adaptive immune responses [181]. This included a cohort of 138 patients. Reduced neutrophil CD88, reduced monocyte HLA-DR and elevated proportions of Tregs were all found to be associated with subsequent infection. The presence of immune dysfunction was linked to a commensurate increase in risk of infection, from 14% for patients with no dysfunction to 59% for patients with dysfunction of all three markers [182]. This study demonstrated the feasibility of standardized flow cytometry from multiple sites [183].

Sepsis-induced immunosuppression is global process, this can be seen both in the systemic circulation and in specific organs such as the spleen and lung. In a study investigating the immune status at the time of death, rapid post-mortem spleen and lung tissue harvest was performed at the bedsides of 40 patients who died of severe sepsis this was compared with control spleen and lung tissue. To identify potential mechanisms of immune dysfunction, cytokine secretion assays and immunophenotyping of cell surface receptor-ligand expression profiles were performed. Cytokine secretion in sepsis patients was found to be less than 10% of that in controls, independent of age, duration of sepsis, corticosteroid use and nutritional status. Immunohistological staining revealed extensive depletion of splenic CD4, CD8 and HLA-DR cells in sepsis patients as compared with controls. The study concluded that patients who die in ICU following sepsis have biochemical, flow cytometric and immunohistochemical findings consistent with immunosuppression as compared with patients who die of non-septic causes [184].

In critically ill patients, it has been suggested that IAI is best assessed with multiple measurements of mHLA-DR expression over a duration of time rather than at a single time point. It has been shown previously that a persistent value of <8000 mHLA-DR molecules/cell for over two days is associated with increased risk for NI and mortality. Determination of the appropriate threshold levels of mHLA-DR is challenging given that there are several methods for measuring mHLA-DR expression. HLA-DR positive monocyte with a cut-off at 30% for detection of IAI is a non-standardized method. In a recent comparison of the conventional method with a standardized quantitative assay for mHLA-DR using measurement of bound HLA-DR antibodies per cell (mAb/cell) as a method of standardization, it was determined that the previously established cut-off value of 30% mHLA-DR corresponds to approximately 5000 mAb/cell, and 45% mHLA-DR to approximately 8000 mAb/cell [174,185], with the range between 30% and 45% mHLA-DR termed “borderline immunosuppression”. A cut-off value of 8000 mAb/cell has been used by authors in interventional clinical trials [186].

In terms of outcome prediction, the prognostic value of utilizing mHLA-DR to predict mortality in 79 adult patients with severe sepsis has been investigated in a prospective observational study [187]. mHLA-DR levels were measured on days 0, 3 and 7 following admission to the ICU. ΔmHLA-DR_3_ and ΔmHLA-DR_7_ (defined as the changes in mHLA-DR value on day 3 and day 7 respectively) was compared to the value of mHLA-DR obtained on day 0 of admission. The data for 28-day survivors and nonsurvivors were compared. The 28-day mortality in patients grouped by mHLA-DR expression with 30% as a cut-off value on days 0, 3 and 7 showed no significant difference between the groups suggesting that single measurements at these specific time points had little predictive value unless interpreted as part of a temporal trend. Additionally, it was shown that mHLA-DR levels return to normal in less than 7 days in injured patients who have an uneventful recovery, conversely it remains persistently decreased in patients who died or developed secondary infections. A dynamic view of mHLA-DR expression in critically ill septic patients shows that survivors tend to progressively normalize their levels of mHLA-DR [188].

One study aimed to assess the persistence of sepsis-induced immunosuppression by measuring several markers, among them was mHLA-DR, at ICU discharge and 6 months after ICU discharge in patients admitted to the ICU for septic shock [189]. The authors concluded that while immune alterations persist at the time of ICU discharge, there are no ongoing immune alterations in septic shock survivors 6 months later.

The value of temporal changes in mHLA-DR levels in the prediction of mortality has been further demonstrated in studying patients with severe acute pancreatitis (SAP). One group assessed the change in mHLA-DR on survival in SAP patients [190]. Survivors were found to have upregulated mHLA-DR expression whereas in the late mortality group it was persistently downregulated. mHLA-DR expression on day 10 (HLA-DR10) gave the only statistically significant correlation with late mortality. ROC curve analysis confirmed that HLA-DR10 was a reliable predictor for late mortality with AUC of 0.944; The optimal cutoff value was 52.3% with a sensitivity of 94.4% and specificity of 85.7%. In another study of 64 patients with SAP, mHLA-DR expression was measured at admission and 7 and 14 days following the onset of SAP [191]. The study demonstrated that patients with persistently low percentages of mHLA-DR throughout the observation period was more likely to develop sepsis in the clinical course subsequently. It was concluded that this was a reliable predictor of the development of sepsis in SAP patients.

Therefore, introduction of mHLA-DR measurement as a point-of-care test at the bedside in ICU may be beneficial for critically ill patients. An automated tabletop cytometer may be a suitable tool for ICU patients as well as for clinical trials as there is no need for sample preparation nor specific skills in flow cytometry and the results are obtained in less than 30 min [192].

In addition to mHLA-DR expression, an alternative method of assessing immune status that has been extensively investigated involves detection of ex vivo lipopolysaccharide (LPS)-induced TNF-alpha production. This is a functional test of monocytic immune capacity. Recently, a comparison of mHLA-DR expression and ex vivo LPS-induced TNF-alpha production and their effect on 28-day outcome and development of secondary infections predictors in severe sepsis was performed in a prospective observational study of 83 adult septic patients [193]. Blood samples were collected at three time points: days 1–2, 3–4 and 6–8 after the diagnosis of sepsis. The study showed that mHLA-DR expression was significantly reduced in nonsurvivors on days 3–4 and 6–8. Furthermore, median mHLA-DR expression decreased from days 1–2 to days 3–4 in patients who developed secondary infections while it was found to be increased in those who did not. This again suggested that changes in mHLA-DR expression over time rather than values at individual time points would be more useful for prediction of outcome. The study postulated that mHLA-DR expression may not be predictive at an early phase of sepsis because circulating monocytes are likely to be recruited out of the bloodstream to sites of active infection, thus resulting in an underestimation of the magnitude. Ex vivo LPS-induced TNF-alpha production did not differ between survivors and nonsurvivors nor between patients who developed secondary infection and those who did not. There was a statistically significant correlation between LPS induced TNF-alpha production and mHLA-DR expression. The group also noted that studies of LPS-induced TNF-alpha production to date primarily utilized pediatric populations; in light of the increasing recognition of the impact of immunosenescence to blunt host response to infection, it was suggested that the increased age and high incidence of co-morbidities may contribute to a labored TNF-alpha response. The study found mHLA-DR to be a more accurate predictor of mortality and secondary infections. In this particular study, the effect of diabetes mellitus, as a co-morbidity, on immune response in sepsis was not taken into account, it would be interesting to address this in future studies [194].

There may be a link between immunosenescence and the consequent state of immune system that increases risk for a dysregulated inflammatory picture. Elderly patients are known to display enhanced apoptotic pathways that may contribute to the incidence of mortality due to sepsis [195]. Evidence supporting this can be seen in a study of 73 critically ill patients in whom ex vivo LPS-induced TNF-alpha production was measured and found to be similar patients who did and those who did not develop an ICU-acquired infection [196]. A study carried out a decade ago found differing results. The study recruited 19 septic trauma patients [197]. On the day after the clinical diagnosis of sepsis, ex vivo LPS-induced TNF-alpha secretion was found to be significantly lower in nonsurvivors as compared with survivors of sepsis. The study concluded that ex vivo LPS-induced TNF-alpha production may be superior as an early predictor of clinical outcome in multiple trauma patients with sepsis when compared to mHLA-DR expression.

Another consideration to employing mHLA-DR measurements in an intensive care setting is the relative ease of running such a test [198,199]. Future interventional studies aimed at the immune response during sepsis might be able to combine a functional test with a phenotypic immunological biomarker for the purpose of target group selection based on biological plausibility and potential intervention effectiveness.

The validity of monocyte HLA-DR expression as a predictor of early mortality was explored in a recent study of 52 septic patients. Monocyte HLA-DR expression was found to be significantly lower in nonsurvivors at time of diagnosis as compared with survivors and served as an independent predictor of 28-day mortality following sepsis [200].

Another recent study performed by Duggal et al. showed that CD14^+ve^ HLA-DR^dim/low^ monocytes were found to be diminished in patients with poorer outcomes in ICU [201].

In bacterial sepsis, there has been evidence to suggest that there are different mechanisms of the clinical manifestations of Gram-positive and Gram-negative sepsis. Some microbial challenges may determine levels of mediators that damage the infecting microorganism and the host. For example, Lipoteichoic acid (LTA) of Gram-positive bacteria as well as lipopolysaccharide (LPS) of Gram-negative bacteria has been shown to elicit different response from the host [202,203,204,205,206].

In the setting of trauma, the predictive potential of mHLA-DR in 80 trauma patients was explored in one prospective study [207]. Daily measurements of mHLA-DR were performed during the first 4 days following trauma. The lowest expression of mHLA-DR was found to be on day 2. Patients who restored mHLA-DR expression at day 3 appeared to be protected from infections, and those who displayed persistently reduced expression of mHLA-DR appeared to be at greater risk of infection. The ratio of mHLA-DR expression between day 3 and day 2, at a value of below 1.2, was found to be independently associated with the development of sepsis. Early mHLA-DR monitoring may therefore provide information preceding infection, thus allowing targeted prophylaxis with antibiotic treatment. Another interesting study of trauma patients aimed to investigate the release of DAMPs in the early, prehospital, phase and its relationship with immunosuppression and NI [89]. Blood was obtained from 166 adult trauma patients at the trauma scene, emergency room (ER) and serially afterward. Circulating levels of nuclear and mitochondrial DNA, and HSP70 were determined. Immunosuppression was assessed by qPCR analysis of HLA-DRA gene expression and ex vivo LPS-induced cytokine production. The study found that HLA-DRA expression was attenuated directly after trauma and did not recover during the follow-up period, whereas ex vivo cytokine production revealed an anti-inflammatory phenotype as early as at the point of the trauma scene, it was also shown to persist in the days following that. By the time of arrival at ER there was significantly reduced HLA-DR mRNA associated with increased levels of anti-inflammatory IL-10. This is in contrast with the prevailing theory that immune dysfunction follows trauma. The importance of immunosuppression after trauma was alluded to in the observation that an HLA-DR mRNA ratio between day 3 samples and samples obtained in the ER of <1 was associated with an increased rate of NI. Higher concentrations of nuclear DNA were also associated with infections. The study concluded that plasma levels of DAMPs are associated with immunosuppression that is apparent within minutes/hours of trauma, and this profound immunosuppression is associated with increased susceptibility to NI following trauma.

Another study sought to clarify the complex interplay of the immune response to severe trauma. Ten trauma patients with injury severity scores greater than 20 at days 1, 3 and 5 after injury were evaluated [208]. The study found that circulating monocytes percentage significantly increased after injury, possibly due to enhanced cell proliferation. Ex vivo stimulated TNF-alpha production and percentage of circulating HLA-DR positive monocytes were significantly decreased in trauma patients compared with age- and gender-matched controls at all time points. These findings suggested that monocyte behavior was significantly influenced by trauma and may display suppressed antimicrobial function. Surprisingly, monocyte phagocytosis was found to be at baseline function and the oxidative burst was augmented suggesting preservation of their innate antimicrobial functions. The study used single-cell mass cytometry to characterize the phenotype and function of major innate and adaptive immune responses in trauma patients. This was another significant study that can potentially pave the way to individualized risk stratification based on deep immune profiling of critically ill patients [209].

Major surgery can also lead to reduced mHLA-DR expression resulting in adverse outcome. In addition to surgical trauma, other causes of post-surgical immunosuppression may include intraoperative hypotension, increased perioperative release of corticosteroids or catecholamines, as well as the application of anesthetic drugs such as fentanyl. One retrospective randomized controlled trial analysis of 10 post-operatively immunosuppressed patients following esophageal or pancreatic resection demonstrated that innate immunity recovered earlier than acquired immunity during severe postoperative immunosuppression. Among other immune markers, mHLA-DR expression was measured pre-operatively up to day 5 after surgery, it was shown that mean mHLA-DR recovery time was on day 5 post-operation [210].

Another study aimed to describe the immediate immune response to major gastro-intestinal surgery in patients over 45 years old with planned post-operative ICU stay. It was concluded that monocyte dysfunction and features of immune suppression occur frequently following major surgery, contributing to post-operative infection [211].

Almansa et al. evaluated the use of procalcitonin (PCT) with gene expression levels of HLA-DRA to detect sepsis in 154 surgical patients. Multivariate and AUC/ROC analysis showed that the PCT/HLA-DRA ratio was superior to PCT for the purpose of detection of sepsis with AUC of 0.85. It was consequently concluded that combination of PCT with HLA-DRA holds promise as a mode for improving sepsis detection in surgical patients [212].

From this discussion, it can be seen that monocytes play a critical role in the innate and adaptive immune systems, performing phagocytosis and orchestrating antigen presentation as well as cytokine production. Recent research has also shown that the MHC class II antigen presentation pathway in human monocytes differs by subset and is regulated by cytokines as such, there is much to be explored yet [213]. Going forward, it can be envisaged that HLA-DR could form a significant part of any immune dysfunction score in the assessment of sepsis, trauma and other forms of critical illness [214].

Recently, two important studies explored the feasibility of circulating and cell-surface immune biomarkers as predictors of infection in critically ill patients (CAPTAIN and ExPRESS study) drawing contrasting outcomes. The CAPTAIN study was conducted to assess the accuracy of circulating biomarkers to discriminate between sepsis and non-septic SIRS. A difference was shown in MFI HLA-DR on both CD14^+High^ and CD14^+Low^ monocytes between sepsis and non-septic SIRS patients (0.9 vs. 1.5, *p* = 0.05; and 2.9 vs. 4.2, *p* = 0.05 respectively). Additionally, there was statistically significant difference in CD64-Neutrophil-MFI between the two groups (2.6 vs. 1.2, *p* = 0.01 respectively). It was shown that eight biomarkers had an area under the receiver operating curve (ROC-AUC) of over 0.6 with a 95% confidence interval over 0.5. LASSO regression analysis identified C-reactive protein (CRP) and HLA-DRA mRNA as being repeatedly associated with sepsis, and no model was found to perform better than CRP alone in this setting (ROC-AUC 0.76 (0.68–0.84)). It was therefore concluded that circulating biomarkers may not be useful in the detection infection at the early phase of sepsis in ICU patients [215].

The ExPRESS-sepsis cohort study recruited patients presenting to emergency departments (EDs) with suspected acute infection and aimed to evaluate the reliability of leukocyte biomarkers as predictors of sepsis (Sequential Organ Failure Assessment score ≥ 2 at 24 h and/or 72 h following ED presentation). In this multicenter cohort study in four EDs and ICUs, flow cytometry was utilized and patients with suspected acute infection (Group 1) with two comparator cohorts: ICU patients with established sepsis (Group 2), and ED patients without infection or systemic inflammation but requiring hospitalization (Group 3) were compared, and 272, 59 and 75 patients were recruited to cohorts 1, 2 and 3, respectively. Of the 47 leukocyte biomarkers examined, 14 were found to be unreliable, and 17 failed to discriminate between the three cohorts. In group 1, eight neutrophil CD antigens, along with seven monocyte and a T cell lymphocyte antigen were analyzed for their ability to predict consequent sepsis in patients who were suspected of sepsis. Individually, only raised neutrophil PD-1 (OR 1.78 (95% CI 1.23–2.57); *p* = 0.002), raised monocyte PD-1 (1.32 (1.03–1.70); *p* = 0.03) or reduced monocyte HLA-DR (0.73 (0.55–0.97); *p* = 0.03) expression were associated with subsequent sepsis. From a large panel of leukocyte biomarkers, markers of early immune suppression (neutrophil and monocyte PD-1 and PD-L1; monocyte HLA-DR) had the strongest association with clinical outcomes. Increased neutrophil PD-1 and reduced monocyte HLA-DR expression were associated with deterioration to sepsis, suggesting that immune suppression may be an early event, prior to development of sepsis [216].

Myeloid cell responses in sepsis are intertwined and complex. One example relates to the plasticity of these cells, which allows immature neutrophils to undergo differentiation to become monocytic cells [217]. Following sepsis, decreased major histocompatibility complex (MHC) mRNA expressions of class II-related genes have been reported; in one study, mRNA expression of five MHC class II-related genes (CD74, HLA-DRA, HLA-DMB, HLA-DMA, CIITA) were measured by quantitative reverse transcription (qRT)-PCR and monocyte human leukocyte antigen-DR (mHLA-DR) by flow cytometry in septic shock patients [218]. The authors reported that the best prognostic value regarding lethal outcome was obtained for CD74 (HLA-DR antigen-associated invariant chain). They concluded that decreased CD74 mRNA expression significantly predicted 28-day mortality following septic shock. Expression of the MHC class II-related genes HLA-DRA and CD74 was investigated in patients with complicated and uncomplicated *Staphylococcus aureus* bacteremia (SAB) [219]. The complicated SAB group included patients with hematogenous seeding or extension of infection beyond the primary focus, etc. It was reported that patients with complicated SAB show weaker HLA-DRA expression than those with uncomplicated SAB during the first week of bacteremia.

In a different study, HLA-DR expression on monocyte subsets was investigated in critically ill children [220]. This population was compared with healthy children, and it was found that HLA-DR expression significantly decreased within all monocyte subsets, being most manifest on classical monocytes and in patients with sepsis. They concluded that low HLA-DR expression on classical monocytes was associated with NI and lethal outcome. Immune responses were investigated in another specific group of non-neutropenic patients with abdominal sepsis, with a focus on prospective invasive candidiasis (IC) risk prediction based on immune markers, including HLA-DR [221]. The authors found that HLA-DR expression, over the first five days, showed no relevant difference between three groups of patients: with no colonization or IC, with subsequent colonization and with subsequent IC.

Various aspects of monocyte signaling can be assessed as potential sepsis immune markers. A monocyte distribution width value greater than 20.0 U is effective for sepsis detection in the emergency department [222]. Authors of a recent study focused on a novel type of RNA class that is naturally resistant to degradation by exonucleases, termed circular (circ)RNA [223]. They explored patterns of circRNA expression in peripheral monocytes of critically ill patients with sepsis secondary to community-acquired pneumonia relative to healthy donors. The authors concluded that circRNAs were more abundant in immune cells of sepsis patients.

Immune response in context of different causative pathogens and sites of infection is seldomly researched. Our group has investigated various aspects of the immune response to different bacteria, origin of secondary sepsis and outcome [12,203,204,224] for over a decade.

## 6. Neutrophil CD64 Expression

Neutrophils, comprising up to 50–70% of total circulating leukocytes, are effector cells of the innate immune system and act as the first line of defense against infections. An important function of neutrophils is the intracellular killing of bacteria after phagocytosis in the phagolysosome. They have a short life span and do not show proliferative properties. Neutrophils can be rapidly activated with a significant increase in number and play a crucial role in initiating an adequate immune response and controlling the microorganisms, making their monitoring relevant in patients with infections [225]. Critically ill post-surgical, post-trauma and/or septic patients experience significant inflammation. Although neutrophils detect chemotactic gradients and migrate toward the site of infection during severe sepsis, this property is impaired in inflammation due to the “neutrophil paralysis”. Neutropenic patients cannot control infection locally, and the resulting systemic spread of microorganisms is associated with high mortality and susceptibility to nosocomial infections. During sepsis, neutrophils undergo alterations in morphology (size, shape and composition), mechanics (deformability) and motility (chemotaxis and migration) [226].

There are two active anti-microbial mechanisms of phagocytosis with one dependent and the other independent of oxygen. The extent of each mechanism varies depending on tissue perfusion and oxygenation in inflammation. Uncontrolled neutrophil activation in capillary beds can lead to organ damage through excessive degranulation in extracellular space and release of lytic proteases; a vicious cycle can form as tissue damage attracts more activated neutrophils. Neutrophil extracellular traps (NETs) consist of fibers made of DNA, chromatin and granules with antimicrobial enzymes, e.g., myeloperoxidase and elastase. NETs formation is mainly due to LPS, TNF-alpha and IL-8, and neutrophils die during the process by “NETosis”. Some anuclear neutrophils however are viable after NETs formation and intravital microscopy has revealed that they remain capable of phagocytosis. In an LPS-induced endotoxic shock model, NETs have shown the ability to adhere to and activate vascular endothelium. During sepsis, interactions between activated platelets and neutrophils induces NETs formation that contributes to endothelial cell damage, tissue destruction and organ injury [227].

In acute inflammation, neutrophil function may be impaired and immature or banded cells with suboptimal microbicidal activity may be released in systemic circulation. Neutrophils can also affect other immune cells paralyzing the adaptive immune system. This immunomodulation encompasses T cells and macrophages that, after phagocytosis of apoptotic neutrophils, shift toward releasing anti-inflammatory cytokines. Although sepsis increases circulating neutrophil numbers, the percentage of immature cells increases, as does cell size and stiffness. This is accompanied by a decrease in migration/chemotaxis. In summary, neutrophils have a paradoxical beneficial and detrimental role in sepsis [228,229,230].

One of the most consistent and profound alterations in septic neutrophils is their activation of a survival program that resists constitutive tendency of the neutrophils to die an apoptotic death following its release from the bone marrow [231]. While around 50% of resting neutrophils will undergo apoptotic morphologic changes following 24 h of in vitro culture, only 5% to 10% of septic neutrophils will have the same fate [232].

CD64 (Cluster of Differentiation antigen 64) is a type of integral membrane glycoprotein known as an Fc fragment receptor I that binds monomeric immunoglobulin G-type antibodies with high affinity (FcgammaRI). Neutrophil CD64 (nCD64) appears to be a marker of neutrophil activation in systemic acute inflammatory response as its expression starts from less than 1–2000 sites per cell at a resting state and becomes gradually upregulated depending on the intensity of stimulation. Within 4–6 h it can reach more than 10-fold higher levels contrary to monocytes with constitutively expressed CD64 antigen [233]. Neutrophil CD64 index is designed such that normal inactivated neutrophils yield values of <1.00 and blood samples from individuals with documented infection or sepsis typically show values >1.50. Quantitative nCD64 expression can be used as a biomarker of bacterial infection.

An observational prospective study of 293 critically ill patients investigated whether the CD64 index differentiates bacterial sepsis from viral and fungal sepsis [234]. The study showed that CD64 index greater than 2.2 predicted bacterial infection (AUC 0.80, sensitivity 63%, specificity 89%). The CD64 index was statistically significantly higher in severe sepsis/septic shock (3.7) than in sepsis (1.5) or SIRS (1.0). This biomarker was also significantly higher in nonsurvivors (2.0) compared with survivors (1.5). Other studies showed that the nCD64 index can be lower in Gram-positive infections than in Gram-negative infections [235], and that it can rapidly diminish after initiation of tailored antibiotic therapy [236].

Gibot et al. enrolled 300 consecutive patients to construct a biologic score that was later validated in an independent prospective cohort of 79 critically ill patients from another center in a landmark study on the usefulness of combination biomarkers to diagnose sepsis [237]. These biomarkers included plasma concentration assays of soluble triggering receptor expressed on myeloid cells-1 (sTREM-1) and procalcitonin (PCT), and nCD64 expression index in flow cytometry. A bioscore was constructed combining these biomarkers. All biomarkers were significantly higher in septic patients compared with patients without sepsis and were independent predictors of sepsis in multiple logistic regression analysis. Although all biomarkers were independent predictors of infection the best ROC/AUC was seen with the nCD64 index with the highest discriminative value (AUC 0.95, specificity 95.2%, sensitivity 84.4%). The nCD64 index was 1.51 (0.98–3.05) in all patients, 0.99 (0.84–1.26) in patients without sepsis and 2.99 (2.04–4.79) in septic patients with the difference statistically highly significant.

Immune markers after major surgery are generally elevated and fall within two days of the procedure. A study on 229 patients following major colorectal, maxillofacial and open heart surgery investigated the expression of nCD64 [238] and found higher nCD64 expression levels in patients with postoperative infections. This biomarker predicted postoperative infection immediately after surgery (AUC 0.902, sensitivity 86.6%, specificity 92%), 24 h after the procedure (AUC 0.891, sensitivity 91.0%, specificity 79.0%) and 48 h after the procedure (AUC 0.823, sensitivity 82%, specificity 78%).

A meta-analysis comprising eight studies and 1986 patients investigated the nCD64 expression as a diagnostic marker for adult sepsis with a pooled sensitivity of 76%, specificity of 85% and AUC of 0.95 [239].

In our previous study of 102 critically ill severe sepsis and/or trauma patients with MODS, we aimed to assess the prognostic value and daily trend of IL-6, nCD64 expression, CRP and lipopolysaccharide-binding protein (LBP) in relation to the outcome measure of hospital mortality. Blood samples were collected on admission (day 1), days 2 and 3. We found that the CD64 index was 1.6-fold higher on the day 1 and 1.78-fold higher on the day 2 in nonsurvivors (*p* < 0.05). The AUC for the CD64 index on day 1 for outcome was 0.727. At a cut-off level of 2.80, sensitivity was 75% and specificity was 65%. Patients with a CD64 index level on day 1 of higher than 2.80 had 2.4-fold higher probability of dying. In our study the CD64 index on day 1 was a fairly good predictor of outcome. AUCs for the IL-6, CRP and LBP were <0.55, and these biomarkers failed to predict outcome [224].

A major challenge in the clinical use of nCD64 expression is the lack of an accurate and rapid point-of-care device. One study investigated a microfluidic biochip for nCD64 expression quantification from a small whole blood sample of 10 microliters without any manual processing [240]. The disposable biochip returns results in less than 30 min. The device was tested on 450 samples from SIRS-positive patients. Among 68 sepsis survivors, it was found that their nCD64 expression increased and then decreased, whereas this count continued to rise until death in the six nonsurvivors. Results from the biochip correlated with those from hematology analysis and flow cytometry. Possible limitations of using this biomarker for diagnosis and prognosis include Gram-positive infections or in patients receiving antibiotics [241].

In another study, flow cytometry was utilized in the prediction of early clinical deterioration and overall survival in 781 patients with confirmed sepsis admitted in the ED and ICU. Several immune markers were investigated, among them was CD64 expression. It was found that on diagnosis, patients with sepsis exhibited increased levels of CD64^pos^ granulocytes. CD64 expression was statistically significantly higher in patients who had sustained an acute kidney injury on day 2 (*p* < 0.001) but was not associated with a worsening of sepsis on day 2. The group consequently concluded that early flow cytometric measurements of CD64^+ve^ granulocytes could help clinicians to target patients at high risk of clinical deterioration [242]. In one study, expression of CD64 on neutrophils was specific for discriminating patients with sepsis but showed weak sensitivity [243].

In a recent study, authors aimed to test whether 24/7 point-of-care analysis of neutrophil marker expression by automated flow cytometry can be achieved after polytrauma [244]. They demonstrated that polytrauma patients, who developed secondary infections, had significantly higher %CD16^dim^/CD62L^bright^ neutrophils compared with those who did not develop infectious complications; AUC value was 0.90, which is excellent.

## 7. Monocyte CD64 Expression

Constitutive monocyte CD64 expression has been studied to a lesser extent in comparison with expression of this high-affinity and restricted isotype-specificity FcγRI receptor on neutrophils in sepsis. It is to be expected that prominent expression of CD64 on neutrophils would be accompanied with augmented expression of CD64 on monocytes in this clinical setting [245,246]. In one study authors demonstrated that, at the onset of sepsis, expression of CD64 on both neutrophils and monocytes was similar. Yet, after 24 h, diagnostic accuracy of CD64 on monocytes was significantly lower in comparison with neutrophils [247]. Another group of authors introduced simultaneous quantitative analysis of CD64 expression on both types of cells as an improved way to detect infections, designated “CD64 score point” [248]. Danikas et al. investigated whether higher monocyte CD64 expression is associated with increased phagocytic activity and outcome in patients with sepsis [249]. Authors demonstrated that lower phagocytic activity and monocyte CD64 expression were associated with worse outcome. In another study regarding neonatal sepsis, authors evaluated expressions of CD64 on monocytes, lymphocytes and neutrophils with flow cytometry to calculate CD64 ratios to be used as a supplementary diagnostic tool [250]. Interesting study focused on immunophenotyping of monocytes during human sepsis demonstrated increased monocyte CD64 expression, a receptor related to phagocytosis, in patients with sepsis [251]. Preserved phagocytic activity as well as elevated monocyte CD64 expression might be indicators that monocytes are not anergic in septic patients, with higher levels in survivors. In a recent study with a small sample size, authors did not find alterations of monocytes in patients with sepsis. in addition, they reported that monocyte CD64 expression was not related to mortality risk in this patient population [243]. This is in accordance with our results (unpublished data) from our investigation of 86 critically ill patients with secondary sepsis. We studied a broad panel of immune biomarkers on neutrophils and monocytes, among them was CD64 on monocytes. There was no statistically significant difference between survivors and nonsurvivors in monocyte CD64 expression, neither on the first nor on the fifth day. In both groups there was the same trend between these time intervals: monocyte CD64 expression was statistically significantly higher on the first day in comparison to fifth day (in survivors *p* < 0.01, in nonsurvivors *p* < 0.05). We found statistically significant correlation of monocyte CD64 expression with origin of secondary sepsis, i.e., underlying condition: in patients with peritonitis expression of CD64 on monocytes was the lowest in comparison with pancreatitis or trauma patients.

Monocyte CD64 expression was also investigated in patients with severe COVID-19. High CD64 expression on monocytes was detected both in children [252] and adults [253] with severe SARS-CoV-2 infection; thus, it seems that this is characteristic feature of severe form of COVID-19.

## 8. Immunopathogenesis of Coronavirus Disease 2019—COVID-19

Severe acute respiratory syndrome coronavirus 2 (SARS-CoV-2) is the fifth pandemic in this century (World Health Organization—WHO declared the status of a pandemic threat on 11 March 2020); and is the third human coronavirus epidemic following SARS-CoV in 2002 and the Middle East Respiratory Syndrome coronavirus (MERS-CoV) in 2012 [254]. SARS-Cov-2 is the causative pathogen of coronavirus disease 2019 (COVID-19) and belongs to the *Coroviridae* family; it is a single-stranded RNA (ss-RNA) virus with the largest genome among RNA-viruses with a length of 29,903 nucleotides. The virus is coated with prominent crown-like proteins on its surface that induces immune responses. Similar to the two other viruses, SARS-CoV and MERS-CoV, this one also utilizes angiotensin-converting enzyme (ACE) 2 as a receptor to enter target cells. Host defenses start with an innate immune response which is responsible for both viral control and tissue damage. Adequate production of interferons (IFN) (types I and III) promotes intracellular antiviral defenses in cells targeted by viruses. Cytokine storm has been the focus of severe COVID-19. Authors of numerous studies, at the beginning of the pandemic, reported that serum levels of various cytokines were higher in ICU COVID-19 patients as well as in nonsurvivors [255,256,257]. Several studies described COVID-19-associated cytokine storm in divided stages. The first stage involves temporary immunodeficiency, and the second stage involves an overactive immune response (cytokine storm) as a compensation for failed viral clearance [258]. High levels of expression of IL-1B, IFN-γ, and other proinflammatory mediators have been detected in patients with COVID-19. These cytokines may stimulate the production of T helper type 1 (Th1) cells. However, individuals with COVID-19 have higher levels of cytokines generated by Th2 cells (such as IL-4 and IL-10), which act as anti-inflammatory agents [259]. Cell and animal models of SARS-CoV-2 infection revealed a unique and inappropriate inflammatory response defined by low levels of type I and III IFNs (IFN α/β) early on, with elevated pro-inflammatory mediators from macrophages at later time points [260]. Therefore, reduced innate antiviral defenses are linked to inflammatory cytokine overproduction. Authors of a recent study performed an integrated immune analysis on a cohort of 50 COVID-19 patients with various disease severity [261]. They described a unique phenotype in severe and critical patients with severely compromised IFN type I responses (no IFN-β and low IFN-α production and activity), which was associated with a sustained blood viral load and an inflammatory response that was significantly expressed (TNF-α and IL-6). The clinical course of COVID-19 is often of the subacute nature which involves possible immunosuppression, due to T cell depletion and exhaustion that contributes to continuous viral load and a lethal outcome [262]. In patients who develop ARDS and/or MODS later in the clinical course of COVID-19, there is a rationale for pro-inflammatory (notably IL-6) cytokine blockade in order to contain the tissue damage. Yet, severe lymphopenia and T cell exhaustion may worsen the condition of the COVID-19 patients who are treated with IL-6 blocking agents. One study demonstrated that both lung-resident and circulating T cells from COVID-19 patients upregulate markers of T cell exhaustion, including PD-1 [263]. T cell counts were found to be reduced significantly in COVID-19 patients, and the surviving T cells appear functionally exhausted. Leukocytopenia, particularly lymphopenia (partly due to direct viral killing of lymphocytes), is a typical finding in COVID-19 cytokine storm [264].

SARS-CoV-2, as a cytopathic virus, induces death and injury of virus-infected cells and tissues as a part of the virus replicative cycle [265]. The healthy respiratory system is rich in ACE2 receptors, expressed on the surface of airway and alveolar epithelium, vascular endothelium and resident macrophages. After being infected, all these host cells are subjected to pyroptosis and can release massive quantity of DAMPs, triggering systemic hyperinflammation. Several routine laboratory parameters have important role in monitoring patients with severe COVID-19. Marked coagulopathies with thrombotic complications are associated with immune dysregulation; therefore, the clinical importance of elevated D-dimer levels is obvious. High circulating LDH levels is a marker of intense pyroptosis. One study showed that low serum albumin levels were associated with increased mortality in COVID-19 patients [266]. Serum hyperferritinemia might also be suggestive of cytokine storm [267]. Ferritin is a major mediator of immune derangements; excessively elevated levels exhibit proinflammatory features and are much higher in COVID-19 nonsurvivors [268]. One meta-analysis showed that a baseline serum ferritin level exceeding 500 ng/mL serves as a prognostic marker of severe and lethal COVID-19 and is an independent risk factor for bilateral lung infiltrations [269]. Lymphopenia in COVID-19 may be a consequence of SARS-CoV-2-induced apoptosis of lymphocytes and/or their depletion due to recruitment to affected organs such as lungs [270,271]. Authors of one study reported a decrease in monocyte, eosinophil, and basophil numbers in these patients [272]. In COVID-19 patients with high inflammatory cytokines, post-mortem pathology findings revealed tissue necrosis and interstitial macrophage and monocyte infiltrations in the lung, heart, and gastrointestinal mucosa [273]. Authors of a review compared severe COVID-19 and sepsis [274]. They concluded that there are striking similarities between patients with severe COVID-19 and those with sepsis, including a dysregulated host response, inappropriate activation of the coagulation cascade, MODS, high mortality, therefore, severe COVID-19 is due to sepsis caused by SARS-CoV-2.

Altered cytokine levels in COVID-19 patients have studied from the beginning of pandemic [275]. “Cytokine storm” is a widespread syntagm both in the scientific literature and in the media. A large study that enrolled 1500 hospitalized COVID-19 patients, demonstrated that serum levels of IL-6, IL-8, and TNF-α were elevated at the time of admission and correlated with mortality. The study led to suggestions that patients with high IL-6 and TNF-α levels should be assessed for combinatorial blockade of pathogenic inflammation in this disease [276]. Therapeutic options proposed for COVID-19 include among others, cytokine storm blockade by using the IL-1 receptor antagonist anakinra or IL-6 receptor inhibitor tocilizumab [277]. Yet, the clinical results of IL-6 blockade with siltuximab or tocilizumab are varied in COVID-19 patients. Given the fact that the viral infection is activating macrophages, suppressing innate and adaptive immunity might lead to dissemination of SARS-CoV-2. Authors of a randomized, double-blind, placebo-controlled trial involving 243 patients with confirmed severe SARS-CoV-2 infection reported that tocilizumab was not effective for preventing intubation or death in moderately ill hospitalized patients with COVID-19 [278]. An important point regarding cytokine storm was made by experts who challenged that this term should be synonymous with pathophysiology of COVID-19 [279]. The term “cytokine storm” is devoid of definition, yet it implies that the levels of produced cytokines are detrimental to host cells. It has been noted that the majority of mediators implicated in cytokine storm exhibit pleotropic downstream effects and are frequently biologically interconnected. Additionally, these mediators’ interactions are neither linear nor uniform. Additionally, they noted that median IL-6 levels are 10- to 200-fold greater in patients with the hyperinflammatory phenotype of ARDS than in individuals with severe COVID-19. They concluded that the term “cytokine storm” may be misleading in heterogenous populations of patients with COVID-19 ARDS and that the linkage of cytokine storm to COVID-19 may be nothing more than a “tempest in a teapot”—an interesting idiom used by the authors. One preliminary study compared TNF-α, IL-6 and IL-8 levels in critically ill COVID-19 vs. non-COVID-19 patients [280]. They included critically ill patients with following conditions: COVID-19 with ARDS, sepsis with ARDS, sepsis with no ARDS, out-of-hospital cardiac arrest (OHCA) and trauma. It was revealed that levels of all three cytokines were significantly lower in COVID-19 patients than in patients with bacterial sepsis with or without ARDS. They concluded that critically ill patients with COVID-19 with ARDS had lower circulating cytokine levels compared with patients with bacterial sepsis and were similar to those seen in other critically ill patients (OHCA, trauma). Another preliminary study indicated that, while mHLA-DR expression levels were lower in COVID-19 patients than in healthy donors, the degree of immune suppression was less severe than in patients with bacterial septic shock [281]. They concluded that there is more moderate innate immune suppression in COVID-19 patients compared with bacterial septic shock patients; this is in accordance with a low incidence of secondary infections in COVID-19 patients. In this study, none of the patients developed a secondary infection during the follow-up period of 16–17 days post-ICU admission. In line with above-mentioned studies, a recent review focused on lung-centric COVID-19 macrophage activation [282]. Severe SARS-CoV-2 infection leads to intra-pulmonary immune activation, including regional but not systemic macrophage activation with associated immunothrombosis that has been termed pulmonary intravascular coagulopathy (PIC), in contradistinction to disseminated intravascular coagulopathy (DIC). This lung-centric cytokine dysregulation might not trigger considerable elevations in cytokines or systemic inflammatory markers. This is another example of immune response compartmentalization. The authors of the review stated that severe lung-specific and mostly lung-originating cytokine dysregulation reflects “local cytokine flooding” rather than a global cytokine storm.

Severe COVID-19 is marked by a dysregulated myeloid cell compartment. In mild COVID-19, one study indicated that HLA-DR^hi^CD11c^hi^ CD14^+^ monocytes with an IFN-stimulated gene signature were raised using a combination of single-cell RNA sequencing and single-cell proteomics [283]. In the severe stage of the disease, malfunctioning HLA-DR^lo^CD163^hi^ and HLA-DR^lo^S100A^hi^ CD14^+^ monocytes were observed. in addition, neutrophil precursors, as evidence of emergency myelopoiesis, and dysfunctional mature neutrophils expressing PD-L1 and exhibiting an impaired oxidative burst response were found in patients with severe COVID-19. Spatial as well as temporal compartmentalization of immune response in COVID-19 patients was confirmed in another single-cell transcriptomics study [284]. It was demonstrated that infection with SARS-CoV-2 causes a spatial dichotomy in the innate immune response by suppressing peripheral innate immunity in the face of proinflammatory responses in the lung. Additionally, there is a temporal shift in the cytokine response, from an early but temporary type 1 IFN response to a proinflammatory response at the disease’s later and more severe phases. Unexpectedly, enhanced levels of bacterial DNA and LPS (probably of lung origin) can also be found in the plasma of COVID-19 patients, which were positive correlated with augmented release of IL-6 and other inflammatory mediators. In a study conducted in France, one group revealed a high abundance of CD177, a specific neutrophil activation marker, in a cluster of severe COVID-19 patients [285]. Higher levels were confirmed in ICU compared to non-ICU patients. Longitudinal measurements discriminated between patients who died, and those who recovered. These results highlight neutrophil activation as a hallmark of severe disease and CD177 assessment as a reliable prognostic marker for routine care.

Detailed analysis of the features and functional profiles of neutrophils and monocytes in nineteen COVID-19 patients was performed in one study [286]. The analysis showed that neutrophils were unable to upregulate HLA-DR and PD-L1 expression on the surface of immune cells. Neutrophils also display enhanced degranulation of primary granules, and CD10-immature neutrophils were expanded. In the study, IFN signatures were decreased in monocytes but increased in neutrophils. Finally, they demonstrated decreased HLA-DR and increased PD-L1 expression on monocytes. There is currently an ongoing project COVID-IP (COVID-immunophenotyping) that investigates COVID-19 immune signatures. Published preliminary results demonstrated distinct features of immune response in this disease, including IgG overproduction, dysregulated cytokine response, disrupted monocyte and dendritic cell phenotypes and selective cytopenia in T cell subsets [287]. This was identified in a group of 63 hospital-treated patients with COVID-19 who were otherwise highly heterogeneous; all elements of COVID-19 immune signatures were significantly associated with worse prognosis.

In a recently published study, researchers explored inflammatory responses according to COVID-19 disease severity by plasma cytokine measurement and proteomics analysis in 147 COVID-19 patients [288]. Abrogated adaptive cytokine (IFN-γ, IL-17, IL-22) production and prominent T cell exhaustion were seen in critically ill COVID-19 patients, whereas innate immune responses were intact or hyperresponsive. Clustering analysis of differential protein expression demonstrated that patients do not form clusters based on specific inflammatory endotypes. The authors of the study concluded that homogenous hyperinflammatory innate immune responses (higher plasma concentrations of TNF-α and IL-6) in COVID-19 patients are combined with defective adaptive immune responses due to profound lymphopenia, exhausted T cells and decreased functionality. Cytokine release syndrome (CRS) is often loosely referred to as cytokine storm with high production of proinflammatory cytokines (IL-6, TNF-α), and is major mechanism of morbidity and mortality in COVID-19 infection [289]. A study in 71 patients hospitalized with COVID-19 [290] found that levels of IL-6 and IL-10 were significantly higher in critically ill patients with severe COVID-19 compared to those with severe or mild form of disease, and patients with higher levels of IL-10 had shorter overall survival. A recent study reported an atlas of the immune landscape of COVID-19 patients, integrating molecular (single-cell RNA sequencing), functional and clinical data from local sampling through bronchoalveolar lavage and systemic serum sampling [291]. They observed an increase in naïve T cells that displaced memory CD8^+^ T cells in the lung, as well as a decrease in immunological suppression by blood myeloid cells (both monocyte-dependent and neutrophil dependent). The authors hypothesized that a condition of “immune quietness” is associated with severe clinical manifestations and death. They emphasized that the clinical use of antirheumatic medicines, as advocated by those who assert that immunosuppression is a feature of COVID-19, should be limited to mild and severe patients with a favorable prognosis. “Immune silence” could be a result of this cell population’s significant immaturity as a result of an aberrant and skewed myelopoiesis. The authors of this study advocate for the use of medications capable of “reawaken” the host immune system.

Phenomenon of lymphopenia and hypercytokinemia correlated with disease severity and prognosis in various COVID-19 studies. In one research letter, a simplified immune-dysregulation index was proposed to be based on the ratio of IL-6 to lymphocytes count. This was evaluated in 172 COVID-19 patients with overall 28-day mortality rate of 50.5% [292]. The authors found significant associations between lymphocyte, IL-6, IL-6/lymphocyte and 28-day mortality; IL-6/lymphocyte had higher AUC of 0.93 (improved predicted value) compared to IL-6 (AUC 0.88) and lymphocytes (AUC 0.81) alone. According to the value of IL-6/lymphocyte (<15, 15–50 and > 50), patients were divided into three groups where the third group had the highest incidence of 28-day mortality.

Apart from cytokine and genomic storm, there is a phenomenon termed the “lipid storm” in severe COVID-19 patients. One recently published study drew attention to the bioactive lipids that participate in and can modulate prolonged states of inflammation in severe COVID-19 patients [293]. Targeted lipidomic analysis of BALs was conducted in 25 healthy controls and 33 COVID-19 patients requiring mechanical ventilation. BALs from severe COVID-19 patients were characterized by increased fatty acids and inflammatory lipid mediators. There was a predominance of thromboxane and prostaglandins. Leukotrienes were also increased, notably LTB4, LTE4, and eoxin E4. Monohydroxylated 15-lipoxygenase metabolites derived from linoleate, arachidonate, eicosapentaenoate, and docosahexaenoate were also increased. Specific pro-resolving mediators, most notably lipoxin A4 and the D-series resolvins, were enhanced as well, indicating that the lipid mediator storm observed in severe COVID-19 incorporates both pro- and anti-inflammatory lipids.

Immunothrombosis also constitutes an important focus of COVID-19 research. During this process, neutrophils and activated platelets contribute to the formation of a fibrin mesh to trap pathogens alongside the induction of NET formation in the microcirculation. Intravascular NET formation alone can lead to the fibrin-independent occlusion of microvessels, which can be dangerous [294]. Uncontrolled neutrophil activation may lead to an increased vascular permeability due to released neutrophil elastase (NE) and defensins in ARDS, for example. Moreover, NETs may activate alveolar macrophages as well as endothelium, therefore, inflammation is going to be perpetuated [295]. Increased levels of NETs were found in the plasma of COVID-19 patients, and there was positive correlation with disease severity [296]. The concentration of NETs was augmented in plasma, tracheal aspirate, and lung autopsies tissues from COVID-19 patients, and their neutrophils released higher levels of NETs. Compared with controls, COVID-19 patients had higher levels of myeloperoxidase (MPO)–DNA complexes, both in serum and plasma. These complexes are biomarkers of circulating NETs. Additionally, viable SARS-CoV-2 can directly induce the release of NETs by healthy neutrophils. Furthermore, NETs may kill lung epithelial cells in vitro [297]. Immunothrombotic occlusion of pulmonary microvasculature will cause cell death and worsen respiratory function. NE is the principal inducer of immunothrombotic effects is [298]; therefore, targeting this enzyme is valuable therapeutic option [299]. This risk is increased by vasoconstriction induced by cytokine release syndrome observed in severe COVID-19 [300]. Another way to further immunothrombosis is by impaired hypoxic pulmonary vasoconstriction; pulmonary vascular remodeling is hallmark of pulmonary hypertension, possible complication of COVID-19-induced ARDS [301].

Our group investigated myeloid-derived suppressor cells (MDSCs) in critically ill patients with sepsis [204,302]. MDSC-like neutrophils isolated from COVID-19 patients have the capability to inhibit T cell proliferation and IFN-γ release [303,304]. Diminished IFN-γ production is a known feature of COVID-19 infection, and this is possibly caused by these immunosuppressive neutrophils.

In our previous study [203], in almost 400 critically ill patients with sepsis and/or trauma, it was demonstrated that values of neutrophil-to-lymphocyte ratio (NLR) were significantly higher in nonsurvivors. NLR was also found to be a good independent predictor of lethal outcome. As we can consider COVID-19 infection to be a form of viral sepsis, it is not surprising that elevated NLR has emerged as a hallmark of severe COVID-19 [299,305]. It has been suggested that NLR is an independent risk factor and the most powerful prognostic factor for COVID-19 infection severity and outcome [306,307,308,309].

One recently published in-depth study profiled whole blood transcriptomes of three cohorts of COVID-19 patients and 10 controls and conducted data-driven stratification based on molecular phenotype [310]. They discovered that signals linked with neutrophil activation were significantly enriched in severe patient groups. Additionally, comparison of COVID-19 blood transcriptomes to those of over 3100 samples coming from 12 distinct viral infections, inflammatory illnesses, and independent controls revealed highly specific COVID-19 infection transcriptome signatures.

In another interesting study BAL samples were collected from 88 patients with SARS-CoV-2-induced respiratory failure and 211 patients with known or suspected pneumonia from other pathogens and analyzed them using flow cytometry and bulk transcriptomic profiling [311]. They showed that SARS-CoV-2 produces a slowly unfolding, geographically confined alveolitis in which SARS-CoV-2-infected alveolar macrophages and T cells form a positive feedback loop that induces chronic alveolar inflammation. Reduced mHLA-DR expression indicates immunosuppression in critically ill COVID-19 patients in comparison with hospitalized noncritically ill COVID-19 patients [312]. Authors of a recently published study performed longitudinal assessment of immune profile in 64 critically ill COVID-19 patients with ARDS [313]. This group of patients showed persistently low lymphocyte counts and mHLA-DR expression, as well as elevated cytokine levels. The initial increase in type-I IFN response was followed by a rapid decline over time. A significant discovery was that survivors and nonsurvivors displayed apparent similar immune responses throughout the first three weeks following ICU admission. They all gradually reverted to normal cellular marker levels and exhibited a progressive decline in cytokine levels over time. Only plasma TNF-α exhibited a modest increase over time and was significantly higher in nonsurvivors than in survivors. The authors noted that this was accompanied by an extraordinarily high rate of secondary infections in COVID-19 patients who had ARDS. They found that this immunological profile resembled the delayed immunosuppression seen in bacterial sepsis. This study illustrated the difficulty in discriminating between survivors and nonsurvivors for patients that are admitted for longer durations in ICU.

As time goes by, with new data emerging, the focus on pathophysiology of severe COVID-19 infection is shifting to new areas. Growing numbers of investigators emphasize that there is no evidence of “cytokine storm” in these patients. Early in the course of COVID-19, patients can develop profound hypoxemia, but full-blown respiratory disfunction is rare. Although atypical, pulmonary compliance in intubated COVID-19 patients is only slightly decreased. Later in the disease course, some patients will develop a characteristic ARDS phenotype [314]. Endothelial dysfunction as well as intense vasodilatation will contribute to pulmonary shunting. An increase in respiratory dead space can be the result of thrombosis of the pulmonary vasculature. In one study, 25 COVID-19 patients underwent computed tomography pulmonary artery (CTPA) scan for suspected acute pulmonary embolism (APE) [315]. In ten of them, APE was confirmed. Thrombotic microangiopathy may also occur. The pulmonary pathology of early-phase COVID-19 pneumonia shows lung-vascular congestion [316]. Thus, vasculopathy will contribute to significant D-dimer elevations. Immunosuppression, endothelial activation, and direct viral-mediated tissue damage, rather than hyperinflammatory injury, mediate COVID-19-induced organ dysfunction [314]. In line with this are results from a post-mortem study which found no evidence of vasculitis or interstitial inflammation in kidneys. Electron microscopy revealed clusters of coronavirus particles with distinctive spikes in the tubular epithelium and podocytes [317]. The binding of SARS-CoV-2 on ACE-2 on endothelial cells will initiate impaired cytokine paracrine signaling, including both pro- and anti-inflammatory as well as pro-apoptotic mediators [318]. Contribution of chemokine-mediated lymphocyte recruitment and subsequent infection of lymphocytes (these cells also express ACE-2) to immune suppressive phenotype is evident with lymphocyte apoptosis, T cell exhaustion, etc. [272]. Clinically, lymphopenia correlates with mortality. Angiotensin (Ang)-II is metabolized by endothelial ACE-2 to form the vasodilatory and anti-inflammatory angiotensin. In the early phases of COVID-19 infection, ACE-2 consumption by viral entry will result in markedly increased levels of Ang-II, which can be seen in plasma samples from COVID-19 patients [319]. Elevated Ang-II levels are also linearly associated to viral load and lung injury. Other investigators also confirmed that SARS-CoV-2 can directly infect endothelium and cause endotheliitis, with promotion of inflammatory cell accumulation and activation of the coagulation system with significantly elevated D-dimer and risk of development of DIC [320,321,322]. Damaged endothelium has significantly decreased antithrombotic activity compared to normal endothelial cells. Increased cytokine levels (TNF-α for instance) might mediate T cell apoptosis which may explain the negative correlation between cytokine levels and lymphocyte counts in COVID-19 infection; in addition, these proinflammatory cytokines as well as ferritin can activate the endothelium of the pulmonary vasculature. We should also consider the mechanism of COVID-19 immunopathogenesis related to the antibodies produced by immune cells. If these antibodies are unable to completely neutralize the virus, virus-antibody complexes can bind to Fc or other receptors on host cells, thereby facilitating virus invasion. This is termed antibody-dependent enhancement (ADE) [15]. Inflammatory responses in COVID-19 may be caused by overactivation of macrophages mediated by ADE, particularly in lungs. This can promote pro-inflammatory polarization of macrophages and release of cytokines leading to tissue damage [323]. Viral injury, dysregulated cytokine release and DAMPs induce localized microvascular inflammation, which triggers endothelial activation, leading to vasodilatation and a pro-thrombotic state [314]. COVID-19 can therefore be viewed as a vascular disorder as well. This has significant therapeutic implications. For instance, modes of early invasive mechanical ventilation should be chosen with great caution in order to avoid additional injury of lung tissue.

One review clearly explained the terms immunothrombosis and thromboinflammation [324]. Immunothrombosis is an intrinsic innate immunity effector pathway triggered by pathogens and injured cells in order to reduce dissemination and survival of invading pathogens. It is primarily initiated by neutrophils and monocytes and is facilitated by the production of microthrombi in areas of the microvasculature exposed to pathogens. Activation of innate immunity will lead to tissue factor (TF) and NETs release. Histones from NETs can directly activate platelets and dose-dependently enhance thrombin generation. Conversely, platelets will bind directly to neutrophils, NETs, as well as pathogens. Furthermore, these cells will promote immune cell accumulation. Microorganisms fight immunothrombosis with release of streptokinase (to dissolve fibrin) and/or nuclease (to degrade NETs). However, when immunothrombosis is uncontrolled, it will lead to unregulated activation of the coagulation system. Result is formation of microthrombi and inflammation; when thrombosis is developed, which is referred to as thromboinflammation. Frequently, DIC accompanies this process. It has been proposed that exaggerated immunothrombosis within the pulmonary microvasculature, along with systemic viraemia early in the disease are crucial for the clinical manifestation of COVID-19 infection [324].

COVID-19-associated endothelial dysfunction can be described as lung-centered injury primarily affecting the vascular endothelium. SARS-CoV-2 exhibits tropism for ACE-2 expressed by type II pneumocytes which are anatomically close to lung vasculature [325]. In one interesting study, endotheliopathy and coagulopathy were investigated in COVID-19 patients [326]. Higher levels of soluble P-selectin, marker of both endothelial and platelet activation, were seen in ICU in comparison with non-ICU patients. Similarly, higher levels of thrombomodulin, a specific marker of endothelial activation released during injury of endothelium, was associated with mortality. Higher numbers of circulating endothelial cells were found in severe COVID-19 patients [327]. Endothelial dysfunction in COVID-19 infection is confirmed in a recently published study [328] that found higher plasma levels of both soluble intercellular adhesion molecule-1 (sICAM1) and vascular cell adhesion molecule-1 (sVCAM1) in COVID-19 patients. Apart from endotheliopathy, platelet activation within small blood vessels is increased in COVID-19 patients and it is associated with mortality [329]. The term “microvascular COVID-19 lung vessels obstructive thromboinflammatory syndrome—MicroCLOTS” has been suggested to describe this clinical manifestation [330]. In predisposed people, it has been postulated that alveolar viral damage results in an inflammatory response and microvascular pulmonary thrombosis. This progressive endothelial thrombo-inflammatory disease may potentially affect the brain’s microvascular bed and other important organs, resulting in MODS and death. Endothelial dysfunction and alveolar cell injury both intensify when the ventilation–perfusion mismatch worsens. Additionally, both serum and plasma from COVID-19 patients stimulate healthy neutrophils to generate NETs in a significant way [331]. Apart from immunothrombosis and thromboinflammation, there is another key player, namely the activated complement system. Regardless of pathway of activation (classic, alternative or lectin), the common pathway is responsible for C3a and C5a production, then for stimulating C5b-9 membrane attack complex (MAC) production [324]. This complement attack complex will cause pathogen cell lysis and induces release of procoagulant products and reduce release of natural anticoagulants. C5 will induce TF expression on both leukocytes and endothelium, while C3a and C5a are powerful inducers of proinflammatory cytokine release (particularly TNF-α, IL-1β and IL-6). An interesting viewpoint regarding thrombosis in COVID-19 patients was recently published [332]. The authors discussed how imaging and post-mortem data reveal thrombosis in the pulmonary venous area distal to the alveolar capillary bed, a territory that ordinarily functions as a clot filter and may offer an underappreciated nidus for systemic microembolism. This is consistent with pathological findings of widespread pulmonary venular thrombosis and peripheral organ thrombosis, both of which are associated with pauci-immune cellular infiltrates.

## 9. Can Lessons Learned from Immunopathogenesis of COVID-19 Infection Improve Treatment?

### 9.1. Anticoagulant and Antithrombotic Therapy in COVID-19

Researchers are trying to implement lessons learned from immunopathogenesis of COVID-19 in order to improve therapeutic options. Antithrombotic therapies are widely used. Several trials of heparin and other anticoagulant agents [324] are in progress, these include Randomized, Embedded, Multi-factorial Adaptive Platform Trial for Community-Acquired Pneumonia (REMAP-CAP); the Accelerating COVID-19 Therapeutic Interventions and Vaccines-4 (ACTIV-4) and the Antithrombotic Therapy to Ameliorate Complications of COVID-19 (ATTACC) trial. Investigators participating in the REMAP-CAP study are focused on various elements of COVID-19 treatment among which is evaluating the effect of therapeutic dose anticoagulation using low molecular-weight heparin or unfractionated heparin compared to standard pharmacologic thromboprophylaxis. Results of REMAP-CAP, ACTIV-4a and ATTACC studies were recently published. Numerous investigators concluded that initiating anticoagulation with therapeutic doses of heparin did not result in a higher probability of survival to hospital discharge or a greater number of days without cardiovascular or respiratory organ support in critically ill patients infected with SARS-CoV-2 than did standard-of-care pharmacologic thromboprophylaxis [333]. In parallel, the same group of authors demonstrated that in noncritically ill patients with Covid-19, initiating therapy with therapeutic-dose heparin increased the likelihood of survival to hospital discharge with less reliance on cardiovascular or respiratory organ support than usual-care thromboprophylaxis [334]. In the editorial that accompanied these two publications, possible explanations were raised for different outcomes in different patient populations according to severity of illness. In critically ill patients, the underlying thrombotic and inflammatory damage may have been too advanced to have been influenced by higher doses of heparins. In severe COVID-19, thrombus formation is driven by an orchestra of cytokines, activated complement, platelets, endothelial and inflammatory cells, and microvesicles that provide an efficient catalytic surface for clotting reactions. These surface-bound complexes and fibrin-bound thrombin are quite resistant to inhibition by antithrombin, the key cofactor in heparin and LMWH [335]. Among 75 registered clinical trials of different antithrombotic strategies with different agents in patients with COVID-19, a majority have involved the use of heparin or low-molecular-weight heparin LMWH [336]. The INSPIRATION trial, which compared intermediate doses of LMWH with standard-dose prophylaxis in 562 patients who were being treated in an ICU, showed no between-group difference in thrombotic events, extracorporeal membrane oxygenation treatment, or mortality. They found that there was greater risk of bleeding in the intermediate-dose group [337]. RAPID study researchers gave therapeutic or prophylactic heparin to moderately ill inpatient ward patients with increased D-dimer levels who were admitted for COVID-19. Death, invasive mechanical ventilation, non-invasive mechanical ventilation, or ICU admission were the primary outcomes. Therapeutic heparin had no effect on the primary outcome in this patient cohort, although it did reduce the risk of mortality at 28 days [338]. Several clinical trials of other antithrombotic drugs with different mechanisms of action are ongoing: garadacimab—blockade of factor II; nafamostat mesylate—serine protease inhibitor of thrombin, plasmin and trypsin; tissue-type plasminogen activator—ticagrelor—antiplatelet agent, P2Y_12_-receptor antagonist—attenuation of NET formation [324]. Dipyridamole (DIP) is a phosphodiesterase inhibitor that reversibly inhibits platelet aggregation and potentiates vascular-protective effects of endothelium-derived NO [324]. In vitro, DIP suppresses SARS-CoV-2 replication and improves lung pathology in a model of viral pneumonia. There is a possibility that DIP can prevent NET formation. In a proof-of-concept trial including 31 patients with COVID-19, DIP supplementation resulted in significantly lower D-dimer concentrations, greater lymphocyte and platelet recovery in the circulation, and significantly improved clinical outcomes as compared to control patients [339].

### 9.2. Immunomodulatory Drugs in COVID-19 Treatment

Systemic corticosteroids reduce neutrophils’ respiratory burst and recruitment in sites of inflammation. Dexamethasone is the recommended treatment for severe COVID-19 patients. In a controlled, open-label trial, patients who were hospitalized with COVID-19 were enrolled and randomized to receive either oral or intravenous dexamethasone (at a dose of 6 mg once daily) or standard care alone for up to 10 days. Dexamethasone use resulted in a reduction in 28-day mortality among individuals receiving invasive mechanical ventilation or oxygen alone at the time of randomization, but not among those receiving no respiratory assistance [340].

Another therapeutic possibility is the implementation of anti-inflammatory drugs with different mechanisms of action. Sivelestat is an NE inhibitor that block NET formation and reduce SARS-CoV-2 spike protein proteolytic activation. This drug has been approved in Japan and South Korea to treat ARDS, but with mixed results [324]. Dornase α is a recombinant DNase I that dissolves NETs, and it may help to clear respiratory secretions in COVID-19, thereby reducing risk for secondary infections. Several ongoing trials and studies have shown that COVID-19 patients tolerate dornase α [341,342], but high levels of platelet factor 4 (PF4) in patients with severe COVID-19 infection facilitate binding of PF4 and NETs to form a complex. This complex is compact and resistant to DNase degradation. Combination therapy with heparin, which is known to digest NETs, may be the solution of this problem and allow the drugs to exert systemic effects [324]. Baricitinib is a selective Janus kinase (JAK) inhibitor which prevents activation of the signal transducer and transcription (STAT) pathway, which has systemic proinflammatory effects. Therefore, JAK inhibitors are anti-inflammatory drugs. Baricitinib also inhibits viral entry and impair viral endocytosis; these antiviral effects may also help. In a retrospective, multicenter study, baricitinib reduced COVID-19 mortality rate, as well as ICU admissions in patients with COVID-19 pneumonia. The drug also reduced SARS-CoV-2 viral burden detected by nasopharyngeal swab and was well tolerated by COVID-19 patients [343]. Another JAK 1/2 inhibitor, ruxolitinib is being investigated in an ongoing trial [324]. JAK inhibitors should, to a degree, prevent NET formation. The C5a molecule, which acts on the C5aR1 receptor, was found to increase with increased severity COVID-19 infection [344,345] and promotes neutrophil-induced tissue damage. Thus far, FDA-approved complement inhibitors, eculizumab and ravulizumab, have the same mechanism of action: they bind to C5 and sterically block the cleavage of C5 to C5a and therefore they block MAC formation [324]. Eculizumab (Solris) [346,347] and vilobelimab (monoclonal antibody against C5a, IFX-1) [348] showed promising results in severe COVID-19 treatment. In a case report, a patient was safely and successfully treated with the compstatin-based complement C3 inhibitor AMY-101 [349]. The efficacy of eculizumab has been compared with that of AMY-101, which inhibits C3 cleavage by direct binding, in small independent cohorts of severe COVID-19 patients [350]. Both C3 and C5 inhibitors were observed to induce a substantial anti-inflammatory response, whereas C3 inhibition significantly decreased C3a and sC5b-9 production and inhibited FB consumption. This broader inhibitory profile was associated with a more robust decline of neutrophil counts, attenuated NETs release, faster serum LDH decline, and more prominent lymphocyte recovery. Mesenchymal stem cells (MSCs) are hemopoietic cells that act as immunoregulators. In a pilot study of MSCs transplantation in seven COVID-19 patients, the authors showed that MSCs could significantly improve the functional outcomes of without observed adverse effects [325]. The pulmonary function and symptoms of these seven patients were significantly improved in 2 days after MSC transplantation. Furthermore, the gene expression profile showed that MSCs were both ACE2 and transmembrane serine protease (TMPRSS) -2 negative, which indicated that MSCs are free from COVID-19 infection.

Research is currently focused on COVID-19 and immune checkpoint inhibitors (ICIs) [351]. COVID-19 infection may cause T cell exhaustion with increased PD-1/PD-L1 expression. In this setting, the effect of blockade of this axis with antibodies (like anti-PD1 antibody nivolumab) may restore T cell competence and efficiently counteract the viral infection. Five clinical trials are currently open, to examine the efficacy of anti-PD-1 antibody administration to both cancer and non-cancer patients (four out of five registered studies) affected by COVID-19 [352].

### 9.3. Anti-IL-6 COVID-19 Treatment

Although IL-6 has complex biology and is a cytokine with both pro-inflammatory and anti-inflammatory actions, it recently surfaced as a biomarker of COVID-19 infection early in current pandemic. In 45 patients with severe or critical COVID-19 infection, IL-6 was found to be a good biomarker for earlier detection of COVID-19 severity progression [353]. They found significantly higher levels in critically ill patients (AUC 0.848, sensitivity 90.91%, specificity 66.67%, cutoff value 19.03 pg/mL). In one study with a smaller sample size of 38 COVID-19 patients, it was found that serum levels of IL-6 were significantly higher in nonsurvivors and the optimal cutoff value of IL-6 was 30.95 pg/mL with high sensitivity and specificity as a biomarker [354].

There are three modes of IL-6 signaling: classical receptor signaling, IL-6 trans-signal transduction and trans-presentation. In the classical mode, IL-6 binds to the membrane-bound IL-6 receptor (mIL-6R). In trans-signal mode IL-6 binds to its soluble receptors (sIL-6R). Trans-presentation through juxtracrine signaling is the most recently discovered form of IL-6 signaling. Biological drugs against IL-6 can target the cytokine directly, and include clazakizumab, sirukumab, siltuximab, and olokizumab. mIL-6R is blocked by tocilizumab and sarilumab while sIL-R is blocked by olamkicept, with the latter resulting in the arrest of IL-6 trans-signaling [355]. It should be kept in mind that biological drugs that prevent IL-6 binding to IL-6R will increase concentrations of the cytokine in the systemic circulation. IL-6R blockers can have the same effect to a lesser extent. Although there are studies that imply the possibility of cytokine storm following the blockade of IL-6 signaling, [356] typically, peak IL-6 levels in COVID-19 patients are less than 100 pg/mL. This is significantly less than in the other conditions that we have described above. Tocilizumab (Actemra^®^) is a humanized anti-IL-6R monoclonal antibody. Studies assessing the effectiveness of tocilizumab in the treatment of COVID-19 have shown mixed results and have varied methodologies. These studies have different designs (observational or randomized) and sample sizes and some included parallel comparators to tocilizumab, whereas others do not. Therefore, it is difficult to directly compare the results across these studies. In one study, all 21 patients with severe or critical COVID-19 infection have been discharged on average 15.1 days after administration of tocilizumab. Authors concluded that tocilizumab, which improved clinical outcome immediately in severe and critical COVID-19 patients, is an effective treatment that reduces mortality [357]. In a pilot prospective open, single-arm multicenter study on the off-label use of tocilizumab involving 63 severe COVID-19 patients, an improvement in respiratory and laboratory parameters was observed [358]. Out of 100 COVID-19 patients with ARDS who received tocilizumab twice a day, 77 improved, 23 deteriorated (in this group 20 died) and three had serious adverse events [359]. In another study, administration of intravenous tocilizumab was not associated with changes in 30-day mortality in COVID-19 patients with ARDS [360]. A report of two cases of COVID-19 patients with cytokine release syndrome, who were treated with tocilizumab, demonstrated that progression to secondary hemophagocytic lymphohistiocytosis despite treatment with tocilizumab, and one patient developed viral myocarditis, challenging the safety and clinical usefulness of tocilizumab in the treatment of COVID-19-induced cytokine release syndrome [361]. It is obvious that optimal patient selection and timing for the use of tocilizumab during this disease process is yet to be determined. One randomized, double-blind, placebo-controlled Phase III clinical trial aimed to evaluate safety and efficacy of tocilizumab in patients with severe COVID-19 pneumonia. However, the trial did not meet its endpoints, i.e., improved clinical status and reduced mortality [362]. The same fate has befallen Sarilumab (Kevzara^®^), a fully human Ig-G1 monoclonal antibody that blocks both IL-6R and mIL-R and inhibits classical and trans-signaling. The U.S.-based phase III trial was stopped in 2020 [362]. Siltuximab is a chimeric human-mouse monoclonal antibody against IL-6. Siltuximab was used to treat Severe COVID-19 in the SISCO study. Here, 30 siltuximab-treated patients were matched to 30 control patients who received standard of care treatment alone. Preliminary data showed that patients requiring ventilatory support may benefit from treatment with siltuximab to reduce mortality and cytokine-driven hyperinflammation [363]. Authors of a review [362] pointed out that trans-signaling via sIL-6R may act through proinflammatory pathways and promotes recruitment of innate immune cells and inhibition of T cell apoptosis. However, classic IL-6 transduction has protective, regenerative, and anti-inflammatory effects; thus, they suggest targeting the sIL-6R-dependent trans-signaling pathway alone. A disintegrin and metalloprotease (ADAM) family member, ADAM17 is a type I transmembrane protease that leads to mIL-6R shedding and thus sIL-6R production. Authors of one study concluded that more selective and potent ADAM17 (which is manufacturer of sIL-6R) inhibitors such as A17pro could be more effective and with fewer adverse effects [362].

A meta-analysis of randomized-controlled trials (RCTs) of tocilizumab in COVID-19 infection that used parallel comparators (standard-of-care—SOC), was recently published [364]. Nine RCTs, enrolling 6493 patients, out of which 52.2% in tocilizumab arm, were analyzed [278,365,366,367,368,369,370,371,372]. The study focused on 28-day mortality, which was the primary outcome of interest, secondary outcomes included the need for mechanical ventilation and/or ICU admission. Overall mortality in the tocilizumab group was 24.4% vs. 29% in the control group. Data regarding disease progression showed that, in the tocilizumab group 8.7% of patients required mechanical ventilation and 34.9% were admitted to ICU vs. 10.5% and 41.5% in the control group, respectively. In four of the nine included studies, [278,365,366,367] higher mortality was seen in the tocilizumab group. One of these studies, TOCIBRAS [367] was terminated early because of the increased mortality risk. The RECOVERY and REMAP-CAP trials [371,372], were the most weighed studies in the pooled analysis (75% of all patients). Both of the aforementioned studies reported an effect in favor of the tocilizumab group, i.e., patients who were treated with this drug had lower mortality. Overall, the meta-analysis showed that tocilizumab use may be associated with short-term mortality benefit [364]. Patients in the REMAP-CAP study were the sickest and most benefited from tocilizumab as ICU admission and advanced respiratory support were part of the criteria for recruitment in this study, in contrast to four other studies where these were exclusion criteria. In both the RECOVERY and REMAP-CAP trials, patients who received both tocilizumab and corticosteroids had lower mortality. Data examining those who received only tocilizumab are more consistent with harm; thus, it is possible that an interaction between corticosteroids and tocilizumab exists. There remain unanswered questions regarding the optimal timing and dose of tocilizumab as well as whether therapy should be guided by biomarkers [373]. The beneficial effects of IL-6 are numerous and include increased resistance to infection. There are real concerns that IL-6 blockade might impede immune responses to viral invasion and increase susceptibility to secondary infection in patients with COVID-19 [355,367,374]. Another study showed that high-dose methylprednisolone, followed by tocilizumab, may expedite recovery and lower hospital mortality in COVID-19 patients [375]. In an observational study, out of 860 COVID-19 patients, 589 received steroids, 170 IL-6R antagonists and 101 combination therapy. Authors of this multicenter study observed no differences between the three groups in terms of ventilator-free days and hospital mortality [376]. A total of 10,930 patients participating in 27 trials were included in the most recent meta-analysis that investigated the association between administration of IL-6 antagonists and mortality among patients hospitalized for COVID-19 infection [377]. All-cause 28-day mortality was 21.8% in the IL-6 antagonists’ arm vs. 25.8% in the standard of care (SOC) arm. Tocilizumab was studied in the greatest number of trials, sarilumab was used in several, and siltuximab in one trial. There was no association between IL-6 antagonists administration and increased risk of infection compared with SOC or placebo. The meta-analysis confirmed that significant mortality benefit was only found when IL-6ra were co-administered with glucocorticoids. Antagonists of IL-6 appear to be most effective in hospitalized COVID-19 patients with progressive disease and substantial oxygen requirements; these drugs should therefore not be administered to patients with mild disease nor to patients on prolonged invasive mechanical ventilation [378].

### 9.4. Anti-IL-1 COVID-19 Treatment

Anakinra is a recombinant form (17-kDa recombinant nonglycosylated homolog) of the naturally occurring IL-1 receptor antagonist (IL-1ra) and has a short half-life of 3 to 4 h. It blocks the action of both IL-1α and IL-1β. Anakinra has a good safety profile and a wide therapeutic margin and is used to treat hemophagocytic lymphohistiocytosis (HLH) and other autoimmune diseases [379]. Some COVID-19 patients fulfill the criteria for secondary HLH (sHLH), which include fever, hyperferritinemia (higher than 2000 ng/mL), hepatic dysfunction and coagulation disorder. Authors of one study reported favorable anakinra responses in several severe COVID-19 patients with sHLH [380]. Early use of high intravenous doses of the anakinra in five patients with severe/moderate COVID-19 with pulmonary dysfunction, improved mortality in another study [381]. Some recommend administration of anakinra before ICU admission, in order to prevent sHLH in COVID-19 patients [379]. They also pointed out that anakinra has only mild immunosuppressive effects, and that it does not impair capability to fight bacterial or fungal infections. In contrast to tocilizumab, it targets and inhibits the core element in pathogenesis of sHLH, namely the hyperactive inflammasome loop. Given the fact that IL-1 is powerful inducer of IL-6, anakinra will decrease IL-6 release. In COVID-19-induced damage of endothelium and epithelium, IL-1α is released, and can be blocked by anakinra. The short half-life of anakinra makes it possible to stop fast, in contrast to tocilizumab [379]. The three drugs currently available either block IL-1 from binding to the IL-1 receptor (anakinra) or bind directly to IL-1 (rilonacept and canakinumab) [382]. In one retrospective study, canakinumab was used to treat ten patients with COVID-19 bilateral pneumonia. The drug was safe, well tolerated, and associated with a significant decrease in the level of systemic inflammatory response and an improvement in oxygenation [383]. One systematic review and patient-level meta-analysis of the effect of anakinra on mortality in patients with COVID-19 was published recently [384]. The authors aggregated data on 1185 patients from nine studies [385,386,387,388,389,390,391,392,393] and found that mortality in patients treated with anakinra was significantly lower in comparison to SOC and/or placebo group at 11.1% vs. 24.8%, respectively without an increase in rate of secondary infection. This beneficial effect of anakinra was even more pronounced in patients with CRP levels >100 mg/L or ferritin levels > 1000 ng/mL. Meta-analysis of mortality showed that seven of these nine studies found lower mortality in the anakinra group. In one of the remaining two studies [385] there was no mortality in the anakinra group and in the other [391] there was no statistically significant difference in mortality between groups (anakinra group 19% vs. 18% in control group). Overall analysis showed an overall effect in favor of anakinra administration, which is associated with significantly lower mortality in this meta-analysis. According to two other meta-analyses [364,384], anakinra performed much better in comparison to tocilizumab; the overall effect on mortality was in favor of anakinra is compared to tocilizumab in COVID-19 patients. However, the cohort studies included in the anakinra meta-analysis were not randomized and the overall sample sizes were much lower.

There are several other studies regarding implementation of anakinra in COVID-19 patients that were not included in above-mentioned meta-analysis. In one, investigators retrospectively evaluated and compared 56 patients who received anakinra with a cohort of 56 matched controls. Survival at day 28 was significantly higher in anakinra-treated patients than in the controls (75.0 vs. 48.2%) [394]. In a retrospective cohort study of COVID-19 with ARDS treated with non-invasive ventilation outside of the ICU, administration of high-dose (5 mg/kg twice a day intravenously) anakinra was safe and associated with clinical improvement in 72% of patients [395].

Results of the most recent double-blind, randomized controlled phase 3 trial of anakinra in the early treatment of COVID-19 patients were published. Soluble urokinase plasminogen activator receptor (suPAR) serum levels can signal increased risk of progression to severe disease and respiratory failure in COVID-19 patients. A group of investigators conducted the suPAR-guided anakinra treatment for validation of the risk and early management of severe respiratory failure by COVID-19 (SAVE-MORE) study in order to evaluate the efficacy and safety of early initiation of anakinra treatment in hospitalized patients with moderate or severe COVID-19. Approximately two-thirds of almost 600 patients, with plasma suPAR levels of at least 6 ng/mL, received anakinra, and one-third were placebo controls. Clinical status at 28 days after starting treatment was improved in the anakinra arm while 28-day mortality was lower (3.9% in anakinra arm vs. 8.7% in controls) [396].

Another trial involved 392 COVID-19 patients hospitalized with respiratory dysfunction and hyperinflammatory response; 275 received a placebo, 62 received an IL-1 inhibitor, and 55 received an IL-6 inhibitor (29 received tocilizumab and 26 received sarilumab). Researchers analyzed these three groups and found that inhibiting IL-1, but not IL-6, was associated with a statistically significant reduction in mortality [389]. Combination therapy of anakinra and tocilizumab was investigated in another study. A cohort of 20 patients with COVID-19 pneumonia who received anakinra as salvage therapy after failure of tocilizumab were compared with 20 matched controls in a historical cohort of patients treated with tocilizumab. The study showed that there were no differences in clinical improvement rates at 21 days of follow-up. Moreover, hospital mortality rate for patients receiving anakinra was 55% compared to 45% in the control group. They concluded that treatment with anakinra was not useful in improving the prognosis of patients with tocilizumab-refractory severe COVID-19 [397].

### 9.5. Quality of COVID-19 Publications

COVID-19 research during the pandemic has been challenging, there are many underpowered or duplicated studies with inconclusive results [398]. This is compounded by an “infodemic” of poor-quality medical information [399]. Understandably, there is an urgency to publish information, but without adequate peer review this has led to the publication of studies that ultimately had to be retracted [400]. A solution to this might be through complex platform trials (such as the RECOVERY, REMAP-CAP trials), and gathering of data from international registries. This is particularly important for addressing decisions regarding therapy options. The quality of COVID-19 publications in the top three most cited medical journals (NEJM, Lancet and JAMA) in the early phase of pandemic was found to be significantly lower than non-COVID publications on the level of evidence pyramid. A total of 155 COVID-19 publications were assessed and found to be 18-fold more likely to be of lower quality. It was concluded that the quality of COVID-19 publications in the three highest ranked scientific medical journals was below the quality average of these journals [401].

## 10. Conclusions

This review explores in detail the concept of injury-induced immunosuppression and immuno-inflammatory response and reiterates the need for more accurate functional immune monitoring of monocyte and neutrophil function in critically ill patients with sepsis and/or trauma. In the final week, the REALISM study group published that immune profiling demonstrates a common immune signature of delayed acquired immunodeficiency in patients with various etiologies of severe injury [402]. Immune cells and mediators are understudied in critical care medicine and constitute a difficult area to investigate. A better understanding of the pro- and anti-inflammatory responses’ possible helpful and detrimental consequences can improve the intensive care approach. Clinical outcome may be improved by potential treatment measures. Activating immunity, in that context, is still the focus of extensive research [403,404]. There is a growing body of evidence that identifies the relevant markers that is summarized here. It is clear that for the critically ill, one size does not fit all and that immune phenotyping of sepsis patients may pave the way toward a more personalized approach with tailored therapy for the specific patient [405]. Going forward, these concepts will need to be supplemented with new and improved quantification devices and services in order to provide truly patient-centered care in this setting [406].

COVID-19 infection has been diagnosed in over 259 million patients and has led to over 5.17 million deaths thus far. Its immunopathogenesis is complex. It encompasses a wide spectrum of immune states, from multisystem inflammatory syndrome [407,408] on one end to severe immunosuppression [409] on the other, and everything in between. Each immune state on this scale can cause MODS and death. This emphasizes the complexity of immunotherapies for COVID-19 infection [410]. There is ongoing debate on whether COVID-19 is a form of viral sepsis [411], a new disease entity, with involvement of an endotheliopathy-centered pathogenesis [412], or whether we should consider these to be features of COVID-19-associated sepsis [413]. The pathophysiology of a pandemic that has swept over the world causing millions of deaths remains the focus of intense, in-depth ongoing research. 

## Data Availability

Not applicable.
